# Redescription of poorly known species of *Ceratothoa* Dana, 1852 (Crustacea, Isopoda, Cymothoidae), based on original type material

**DOI:** 10.3897/zookeys.592.8098

**Published:** 2016-05-25

**Authors:** Kerry A. Hadfield, Niel L. Bruce, Nico J. Smit

**Affiliations:** 1 Water Research Group (Ecology), Unit for Environmental Sciences and Management, Potchefstroom Campus, North West University, Private Bag X6001, Potchefstroom, 2520, South Africa; 2 Museum of Tropical Queensland, Queensland Museum; 70–102 Flinders Street, Townsville, Australia 4810

**Keywords:** marine fish parasite, buccal-cavity, mouth, tongue-biter, tongue replacement, Isopoda, Cymothoidae, Ceratothoa

## Abstract

Due to the difficulty in accurately identifying cymothoids, these parasitic isopods are often incorrectly named or confused with other species. Within the genus *Ceratothoa*, a number of recent studies have aimed at clarifying some of the problematic species; however, several of the less studied species still require revision. This paper redescribes, from type material, several poorly known *Ceratothoa* species including *Ceratothoa angulata*, *Ceratothoa capri*, *Ceratothoa carinata*, *Ceratothoa collaris*, *Ceratothoa gilberti*, *Ceratothoa gobii*, *Ceratothoa guttata*, *Ceratothoa italica*, *Ceratothoa oestroides*, and *Ceratothoa verrucosa*, further resolving taxonomic uncertainties within the genus.

## Introduction

Although being one of the physically larger parasitic isopods, cymothoids are still relatively understudied. Often easily observed, these isopods can be located inside the gills, mouths, body cavities and on external surfaces of their fish hosts (Hadfield et al. 2011). Originally, most studies of these parasites were limited to the more populated and accessible regions of the world, such as Europe and North America (Smit et al. 2014). Early taxonomists included the cymothoid isopods in their extensive monographs, but often these accounts were limited in descriptive information. Over the years, several scientists started focusing on this group in more detail and made significant contributions to knowledge on cymothoids. One such notable work is that of Joergen Christian Schioedte and Frederik Vilhelm August Meinert in their series of monographs from 1881 to 1884, where the different life stages, hosts and distributions were all observed (Schioedte and Meinert 1881, 1883, 1884).

However, several cymothoid species have not been studied in many years. A number of factors could be responsible for this lack of research, including a lack of cymothoid specialists, but it is highly probable that many of these species cannot be accurately identified from the original descriptions. This paper revises these poorly known species of *Ceratothoa* Dana, 1852 with redescriptions based on their type material.

Currently there are 30 *Ceratothoa* species known worldwide (according to the World List of Marine, Freshwater and Terrestrial Isopod Crustaceans database (Bruce and Schotte 2016). *Ceratothoa* is one of the more speciose genera within the family Cymothoidae, and is usually found residing in the buccal cavity of the fish host. Recent descriptions incorporate comprehensive descriptions and figures essential for accurately identifying specimens to species level, often absent in many of the original descriptions (Martin et al. 2013, 2015a, Hadfield et al. 2014a, 2014b). These papers have added new species and made several taxonomic changes (bringing species in and out of synonymy) within this genus, but more work is still required for the remaining species. Several of these species are considered questionable or no longer valid due to the lack of type material or inadequate descriptions. Here these lesser known *Ceratothoa* species are revised and updated, separating valid from invalid information and determining their correct taxonomic status where possible.

## Methods

Type material for the *Ceratothoa* species was borrowed where available or drawn at their respective museums. Isopods were processed according to the techniques described in Hadfield et al. (2010, 2013). Species descriptions were prepared in DELTA (Descriptive Language for Taxonomy, see Coleman et al. 2010) using a general Cymothoidae character set. Classification follows that of Brandt and Poore (2003). Host authorities are not included in the text or references; host nomenclature and distribution being sourced from FishBase (Froese and Pauly 2015) and Catalog of Fishes (Eschmeyer 2016).

*Abbreviations*. MCZ – Museum of Comparative Zoology; MNHN – Muséum national d’Histoire naturelle, Paris; NHMUK – Natural History Museum, London; RMNH – Rijksmuseum voor Natuurlijke Historie (Naturalis Biodiversity Center); SAMC – South African Museum, Cape Town; USNM – National Museum of Natural History, Smithsonian Institution, Washington; ZMHB – Zoologisches Museum, Museum für Naturkunde, Humboldt-Universität Berlin; ZMUC – Zoological Museum, University of Copenhagen; TL – total length; W – width.

## Taxonomy

### Suborder Cymothoida Wägele, 1989 Superfamily Cymothooidea Leach, 1814 Family Cymothoidae Leach, 1814

#### Genus Ceratothoa

Taxon classificationAnimaliaIsopodaCymothoidae

Dana, 1852

Ceratothoa Dana, 1852: 303; 1853: 752.—Miers 1876: 104–105.—Haswell 1882: 282.—Schioedte and Meinert 1883: 322–323.—Richardson 1905: 233–234.—Bowman 1978: 217–218.—Brusca 1981: 177–178.—Bruce and Bowman 1989: 1–2.—Horton 2000: 1041.—Martin, Bruce and Nowak 2013: 396; 2015a: 253–254.—Hadfield, Bruce and Smit 2014a: 449–450; 2014b: 3–4.
Codonophilus Haswell, 1881: 471.— 1882: 283.—Hale 1926: 201, 223.
Rhexana Schioedte & Meinert, 1883: 289–290.
Cteatessa Schioedte & Meinert, 1883: 296–297.
Meinertia Stebbing, 1893: 354; 1900: 642; 1910: 103.—Richardson 1905: 236–237.— Menzies 1962: 116.—Schultz 1969: 156.
Rhexanella Stebbing, 1911: 179.
Rhexanella Not Ceratothoa.—Dana 1853: 747.—Richardson 1905: 236.—Schultz 1969: 155.—Kussakin 1979: 287 [= Glossobius Schioedte & Meinert, 1883].

##### Type species.

*Cymothoa parallela* Otto, 1828 (by subsequent designation, see Martin et al. 2015a).

##### Remarks.

Diagnostic characters for *Ceratothoa* include the contiguous and swollen antennular bases, triangular cephalon, and the elongate body (2.1–2.9 times as long as wide). *Ceratothoa* also has a pleotelson and pleonite 1 which are narrower than the other pleonites, and subequal uropod rami which do not extend past the pleotelson. A full diagnosis of the genus is provided by Hadfield et al. (2014b).

#### Ceratothoa angulata

Taxon classificationAnimaliaIsopodaCymothoidae

(Richardson, 1910)

Figure 1

Meinertia angulata Richardson, 1910: 22, fig. 21.
Codonophilus angulatus .—Nierstrasz 1931: 132.
Ceratothoa angulata .—Bruce and Bowman 1989: 2–4, figs 1–2.—Trilles 1994: 116.—Williams, Bunkley-Williams and Pitlik 2000: 157–158.—Paulay, Kropp, Ng and Eldredge 2003: 479.—Ravichandran, Rameshkumar and Trilles 2011: 1–3.—Rameshkumar, Ravichandran and Sivasubramanian 2013: 99–105.

##### Material examined.

*Holotype*. United States National Museum, USA (USNM 41008) – female (21 mm TL; 8 mm W), from Port San Pio, Philippines, near mouth of a small stream, host unknown, 11 Nov 1908 (Richardson 1910).

##### Description.

*Holotype female*. Length 21 mm, width 8 mm.

*Body* oval, twice as long as greatest width, dorsal surfaces smooth and polished in appearance, widest at pereonite 5, most narrow at pereonite 7, lateral margins posteriorly ovate. *Cephalon* 0.5 times longer than wide, visible from dorsal view, triangular. *Frontal margin* rounded to form blunt rostrum. *Eyes* oval with distinct margins, one eye 0.3 times width of cephalon, 0.5 times length of cephalon. *Antennula* more stout but same length as antenna, same length as antenna, with 7 articles. *Antenna* with 7 articles; antennae extending to middle of the eye.

*Pereonite 1* with a slight dorsomedial projection, anterior border straight, anterolateral angles extending to anterior margin of eyes with wide truncated and dorsally projected ridges, slight depression at base of each ridge. Posterior margins of pereonites smooth and straight, with posteroventral angles rounded. Coxae 4–7 rounded; not extending past pereonite margin. Pereonites 1–5 increasing in length and width, 6–7 decreasing in length and width, becoming more progressively rounded posteriorly. *Pleon* with pleonite 1 most narrow, visible in dorsal view; pleonites posterior margin smooth, mostly concave; pleonite 2 not overlapped by pereonite 7; posterolateral angles of pleonite 2 narrowly rounded. Pleonites 3–5 similar in form to pleonite 2; pleonite 5 posterior margin produced medially. *Pleotelson* 0.6 times as long as anterior width, dorsal surface smooth, lateral margins posteriorly narrow, posterior margin subtruncate and shallowly emarginate.

*Pereopod 1* basis 1.6 times as long as greatest width; ischium 0.8 times as long as basis; merus proximal margin with bulbous protrusion; carpus with straight proximal margin; propodus 1.2 times as long as wide; dactylus slender, 1.5 as long as propodus, 3 times as long as basal width. *Pereopod 7* basis 0.8 times as long as greatest width; ischium as long as basis, with a large proximal bulbous protrusion; merus proximal margin with large bulbous protrusion, merus 0.5 times as long as wide, 0.3 times as long as ischium; carpus 0.8 times as long as wide, 1.1 times as long as ischium, without bulbous protrusion; propodus 1.3 times as long as wide, 0.5 times as long as ischium; dactylus slender, 1.7 times as long as propodus, 3.3 times as long as basal width.

*Uropod* same length or slightly longer than the pleotelson; peduncle 1.3 times longer than rami, peduncle lateral margin without setae; rami subequal, extending beyond pleotelson, marginal setae absent. *Endopod* 2.6 times as long as greatest width, straight medial margin, convex lateral margin, apically slightly pointed; *exopod* 2.3 times as long as greatest width, extending to end of endopod, apically rounded.

**Figure 1.**
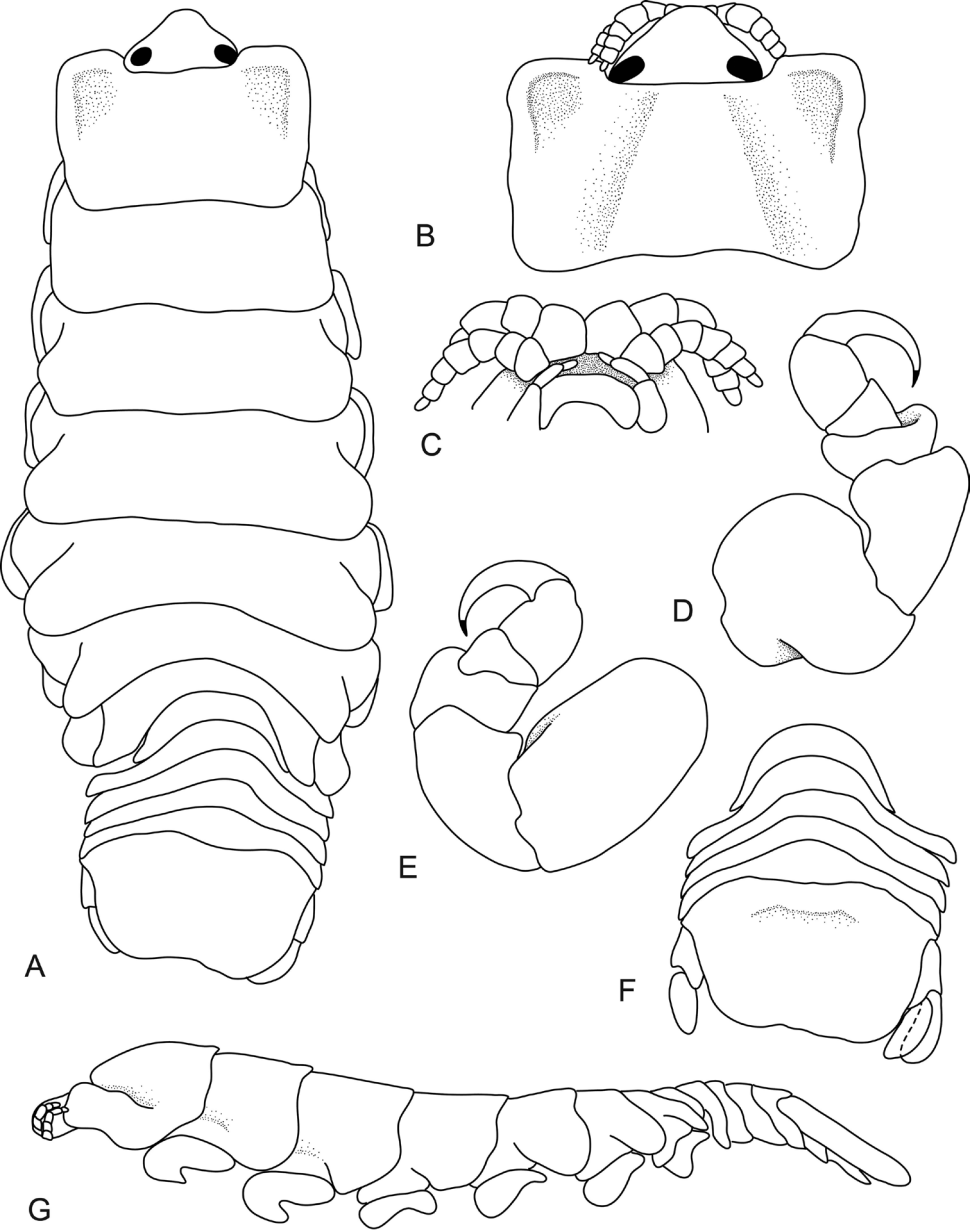
*Ceratothoa angulata* (Richardson, 1910), female holotype (21 mm) (USNM 41008). **A** dorsal view **B** dorsal view of pereonite 1 and cephalon **C** ventral view of cephalon **D** pereopod 1 **E** pereopod 7 **F** dorsal view of pleotelson **G** lateral view.

##### Size.

Female: 17.5–21.5 mm TL (9 mm W); male: 7 mm TL (Bruce and Bowman 1989, Williams et al. 2000).

##### Distribution.

Known from the western and central Indo-Pacific region: Philippines (Richardson 1910); Indonesia (Nierstrasz 1931, Bruce and Bowman 1989); Guam, Micronesia (Williams et al. 2000); and India (Ravichandran et al. 2011, Rameshkumar et al. 2013). The record in Guam extends the range of this species by 2060 km and since this species has only ever been found on the one host species, the isopod range might extend even further as the host has a wider known geographic range in the Indo-Pacific. Ravichandran et al. (2011) recorded this species from India supporting suggestions by Bruce and Bowman (1989) and Williams et al. (2000) that *Ceratothoa angulata* may have a similar distribution to its host.

##### Hosts.

In the buccal cavity of Dussumier’s halfbeak, *Hyporhamphus dussumieri* (previously *Hyporhamphus laticeps*) (Bruce and Bowman 1989, Williams et al. 2000, Ravichandran et al. 2011).

##### Remarks.

The distinguishing characters of *Ceratothoa angulata* include the truncate anterolateral margins of pereonite 1 which form distinct ridges on both lateral sides and two small medial depressions, the slightly emarginate and truncate pleotelson, and the broadly rounded uropodal exopod. The unusually large, quadrate pereonite 1 formed from the lateral ridges is very characteristic for this species.

Richardson’s (1910) description was based on a single specimen, a female from an unidentified host in the Philippines, and consisted of a short description with a single figure. Bruce and Bowman (1989) provided a redescription based on the holotype (with only two figures) and additional material from Borneo (a non-ovigerous female and male), including a short description of the male and figures for both specimens.

*Ceratothoa angulata* resembles *Ceratothoa guttata* with the narrow pleon and pleotelson but the unique pereonite 1 makes it readily distinguishable from other species.

#### Ceratothoa capri

Taxon classificationAnimaliaIsopodaCymothoidae

(Trilles, 1964)

Figure 2

Meinertia capri Trilles, 1964a: 188–198, figs 1–41; 1972a: 1218–1220, figs 219–263, pl. II (17), pl. III (22); 1972b: 1256.—Trilles and Raibaut 1973: 277.
Ceratothoa capri .—Trilles 1986: 623, tab. 1; 1994: 116.—Horton 2000: 1045–1046, figs 5 (c–e).—Rodríguez-Sánchez, Serna and Junoy 2001: 154.—Junoy and Castello 2003: 307.—Kirkim, Kocataş, Katağan and Sezgin 2008: 382–385.—Kirkim, Ozcan and Katagan 2009: 1079–1085, figs 2B & 3B–E.—Innal and Kirkim 2012: A13–A16, figs 1A–B.—Al-Zubaidy and Mhaisen 2013: 166–172, fig. 5.

##### Material examined.

*Lectotype* [here designated]. National Museum of Natural History, Paris (MNHN-IU-2014-17477) – female (16 mm TL, 8 mm W) collected in buccal cavity of *Capros aper* off coast of Nouvelle (Aude, France, Mediterranean), 400–500 m depth, sample (n°81) (Trilles 1964a, 1972a). Also noted: dissected maxilliped, P5–P7 damaged or missing, pleopod 1–2 missing, uropods missing, antennae on one side missing. *Paralectotype*. Male (6 mm TL), same data as lectotype (MNHN-IU-2007-4028) (Trilles 1964a, 1972a).

##### Description.

*Lectotype female*. Length 16 mm, width 8 mm.

*Body* oval, 1.7 times as long as greatest width, dorsal surfaces smooth and polished in appearance, widest at pereonite 5, most narrow at pereonite 1, lateral margins posteriorly ovate. *Cephalon* 0.5 times longer than wide, visible from dorsal view, triangular. Frontal margin rounded to form blunt rostrum. *Eyes* oval with distinct margins, one eye 0.3 times width and length of cephalon. *Antennula* more stout than antenna. *Antenna* with 8 articles.

*Pereonite 1* smooth, anterior border straight, anterolateral angle acute, anteriorly produced, extend to anterior margin of eyes. Posterior margins of pereonites smooth and slightly curved laterally. *Coxae* 2–3 narrow; 4–7 with rounded point; not extending past pereonite margin. *Pereonites* 1–5 increasing in length and width; 6–7 decreasing in length and width; 6 and 7 narrower and becoming more progressively rounded posteriorly. *Pleon* with pleonite 1 most narrow, visible in dorsal view; pleonites posterior margin smooth, mostly concave. Posterolateral angles of pleonite 2 narrowly rounded.

*Pereopod 1* basis 1.7 times as long as greatest width; ischium 0.8 times as long as basis; merus proximal margin with bulbous protrusion; carpus with straight proximal margin; propodus 1.6 times as long as wide; dactylus slender, 1.1 times as long as propodus, 2.9 times as long as basal width. *Pleonites* 3–5 similar in form to pleonite 2; pleonite 5 free, not overlapped by lateral margins of pleonite 4, posterior margin produced medially. *Pleotelson* 0.6 times as long as anterior width, dorsal surface with 2 sub-medial depressions, lateral margins weakly convex, posterior margin subtruncate.

**Figure 2.**
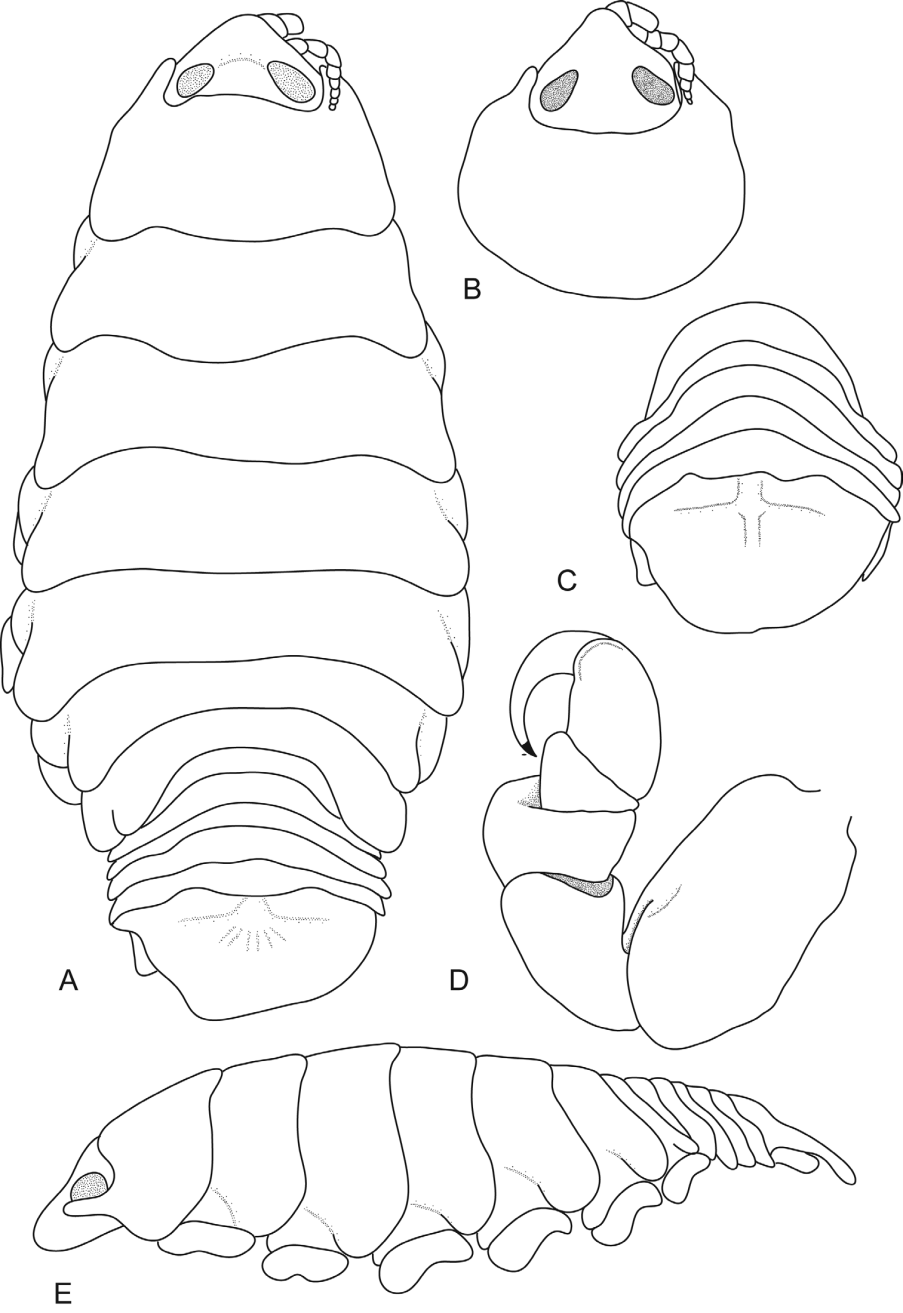
*Ceratothoa capri* (Trilles, 1964), female lectotype (16 mm) (MNHN-IU-2014-17477). **A** dorsal view **B** dorsal view of pereonite 1 and cephalon **C** dorsal view of pleotelson **D** pereopod 1 **E** lateral view.

##### Size.

Female: 13–20 mm TL; male: 6–7 mm TL; second pullus: 2.5–3.5 mm TL (Trilles 1964a, 1972a, b).

##### Distribution.

Throughout the Mediterranean with records from France (Trilles 1964a); Tunisia (Trilles and Raibaut 1973); Straits of Gibraltar and the Alborán Sea (southern Iberian Peninsula) (Rodríguez-Sánchez et al. 2001); Turkey (Kirkim et al. 2008, Innal and Kirkim 2012); Cyprus (Kirkim et al. 2009); and Yemen (Al-Zubaidy and Mhaisen 2013).

Rodríguez-Sánchez et al. (2001) stated that *Ceratothoa capri* was found in the Atlantic and Mediterranean Sea including the Bay of Biscay (Bolívar 1892), Canary Islands (Koelbel 1892) and the Iberian Mediterranean (Barceló Combis 1875, Balcells 1953, Trilles 1977). None of these papers quoted by Rodríguez-Sánchez et al. (2001) mention *Ceratothoa capri*, and it is possible that these were erroneous reference entries for this species. These references were probably intended for *Ceratothoa oestroides*, which is mentioned in each of these articles and was also collected by Rodríguez-Sánchez et al. (2001). Junoy and Castello (2003) repeated this *lapsus* of the references in their checklist for the Iberian Peninsula and Balearic Islands. Rodríguez-Sánchez et al. (2001) also made reference to specific GPS co-ordinates in their paper which appear inaccurate as they correspond to localities on land instead of the expected aquatic points necessary for an oceanographic expedition.

##### Hosts.

On the branchio-spines in the gill and on the bottom of the buccal cavity of *Capros aper* (see Trilles 1964a, 1972a, Trilles and Raibaut 1973); from the buccal cavity of *Boops boops* and *Spicara smaris* (see Kirkim et al. 2008, Innal and Kirkim 2012); *Centracanthus cirrus* (see Kirkim et al. 2009); and *Chelon macroleps* (see Al-Zubaidy and Mhaisen 2013).

##### Remarks.

*Ceratothoa capri* can be distinguished by the acute anterolateral margins which extend past the prominent eyes; body widest at pereonite 5; a narrow pleotelson; and no appendix masculina on the second pleopod in males.

There are a number of species of *Ceratothoa* in the Mediterranean; however *Ceratothoa capri* differs from them all. There are several differences between *Ceratothoa capri* and *Ceratothoa gobii* but the most obvious is the bilobed pleotelson in *Ceratothoa gobii* which is absent in *Ceratothoa capri*. The defining pereonite 1 characters of *Ceratothoa collaris* are absent in *Ceratothoa capri* and differences between *Ceratothoa capri* and *Ceratothoa italica* include less developed eyes, acute and produced anterior margin of the cephalon and the more truncate body of *Ceratothoa italica*. Similar characters separate it from *Ceratothoa steindachneri* as well as the number of articles of the antennae and *Ceratothoa oxyrrhynchaena* differs from *Ceratothoa capri* in the shape of the 7^th^ pereopod basis of the female. Lastly, *Ceratothoa oestroides* is less globular or elliptical when compared to *Ceratothoa capri*; is darker in the post-cephalic region due to more chromatophores; has shorter uropods; and a more stout body.

In the original description of this species, Trilles (1964a) did not designate a holotype; however, full descriptions of the female, male and second pullus were given along with figures of each. Several years later, Trilles (1972a) listed a male and female *Ceratothoa capri* located in the buccal cavity of *Capros aper* from the Gulf of Lion, Mediterranean, which he stated were the types for the species. Examination of these specimens confirms that they are the syntypes of *Ceratothoa capri*. The female specimen is here designated as lectotype and redescribed. This lectotype is necessary to fix and stabilise the identity of this species and use of the name.

#### Ceratothoa carinata

Taxon classificationAnimaliaIsopodaCymothoidae

(Bianconi, 1869)

Figure 3

Cymothoa carinata Bianconi, 1869: 210–211, pl. II, figs 2 (a–b).—Gerstaecker 1901: 258.
Cymothoa (Ceratothoa) carinata .—Hilgendorf 1879: 846.
Ceratothoa carinata .—Schioedte and Meinert 1883: 327–329, pl. XIII (Cym. XX) figs 1–2.—Trilles 1986: 623, tab. 1; 1994: 116–117; 2008: 23.—Kensley 2001: 232.—Bruce 2007: 278.—Trilles 2008: 23.—Martin, Bruce and Nowak 2013: 397–401, figs 1–3.—Nagasawa, Fukuda and Nishiyama 2014: 59–61, fig. 1.—Martin, Bruce and Nowak 2015a: 266–267.
Meinertia carinata .—Lanchester 1902: 378.—Stebbing 1910: 103–104.—Trilles 1972b: 1244–1245, 1256, pl. I, photos 5–7; 1972c: 3–7, photos 1–4.—Avdeev 1979: 48, 50.
Codonophilus carinatus .—Nierstrasz 1931: 132.
Ceratothoa curvicauda Nunomura, 2006: 36–38, figs 12–13.
Ceratothoa sp.—Saito 2009: 7–9, photos 1–2.

##### Material examined.

*Neotype* [here designated]. South African Musuem, Cape Town (SAMC-A085795) – female (33 mm TL; 15 mm W), collected from Maputo Bay, Mozambique, from the buccal-cavity of *Selar crumenophthalmus*, November 2013, coll. Wynand Vlok (HP 221). *Paratypes*. Three females (27–31 mm TL; 12–14 mm W), same data as holotype (SAMC-A085796).

##### Description.

*Neotype female*. Length 33 mm, width 15 mm.

*Body* rectangular, 1.8 times as long as greatest width, dorsal surface with medial longitudinal ridge present, widest at pereonites 3–5, most narrow at pereonite 1, lateral margins slightly convex. *Cephalon* 0.6 times longer than wide, visible from dorsal view, subtriangular. *Frontal margin* rounded to form blunt rostrum. *Eyes* oval with distinct margins, one eye 0.1 times width of cephalon; 0.2 times length of cephalon. *Antennula* more stout and same length as antenna, with 7 articles; antennule peduncle articles 1 and 2 distinct and articulated. *Antenna* with 8 articles.

*Pereonite 1* with median projection, anterior border straight, anterolateral angle with small distinct produced point, extend to middle of the eye. Posterior margins of pereonites smooth and straight. Coxae 2–3 narrow; with posteroventral angles rounded; 4–7 with rounded point; not extending past pereonite margin. Pereonites 6 and 7 narrower and becoming more progressively rounded posteriorly. *Pleon* with pleonite 1 most narrow, visible in dorsal view; pleonites posterior margin with irregular small nodules. Pleonite 2 not overlapped by pereonite 7; posterolateral angles of pleonite 2 narrowly rounded. Pleonite 5 widest, posterior margin produced medially. *Pleotelson* 0.5 times as long as anterior width, dorsal surface with 2 sub-medial depressions, lateral margins weakly convex, posterior margin subtruncate and shallowly emarginate.

*Pereopod 1* basis 1.5 times as long as greatest width; ischium 0.8 times as long as basis; merus proximal margin without bulbous protrusion; carpus with straight proximal margin; propodus 1.7 times as long as wide; dactylus moderately slender, 1.2 times as long as propodus, 2.9 times as long as basal width. *Pereopod 7* basis as long as greatest width; ischium 1.2 times as long as basis, with a large proximal bulbous protrusion; merus proximal margin with large bulbous protrusion, merus 0.5 times as long as wide, 0.3 times as long as ischium; carpus 0.7 times as long as wide, 0.3 times as long as ischium, without bulbous protrusion; propodus 1.3 times as long as wide, 0.5 times as long as ischium; dactylus moderately slender, as long as propodus, 2.2 times as long as basal width.

*Uropod* same length as pleotelson, peduncle 0.8 times longer than rami, peduncle lateral margin without setae; rami not extending beyond pleotelson, marginal setae absent, apices narrowly rounded. *Endopod* apically rounded, 3 times as long as greatest width, lateral margin straight, mesial margin straight, terminating without setae. *Exopod* extending to end of endopod, 4 times as long as greatest width, apically rounded, lateral margin weakly convex, mesial margin straight, terminating without setae.

**Figure 3.**
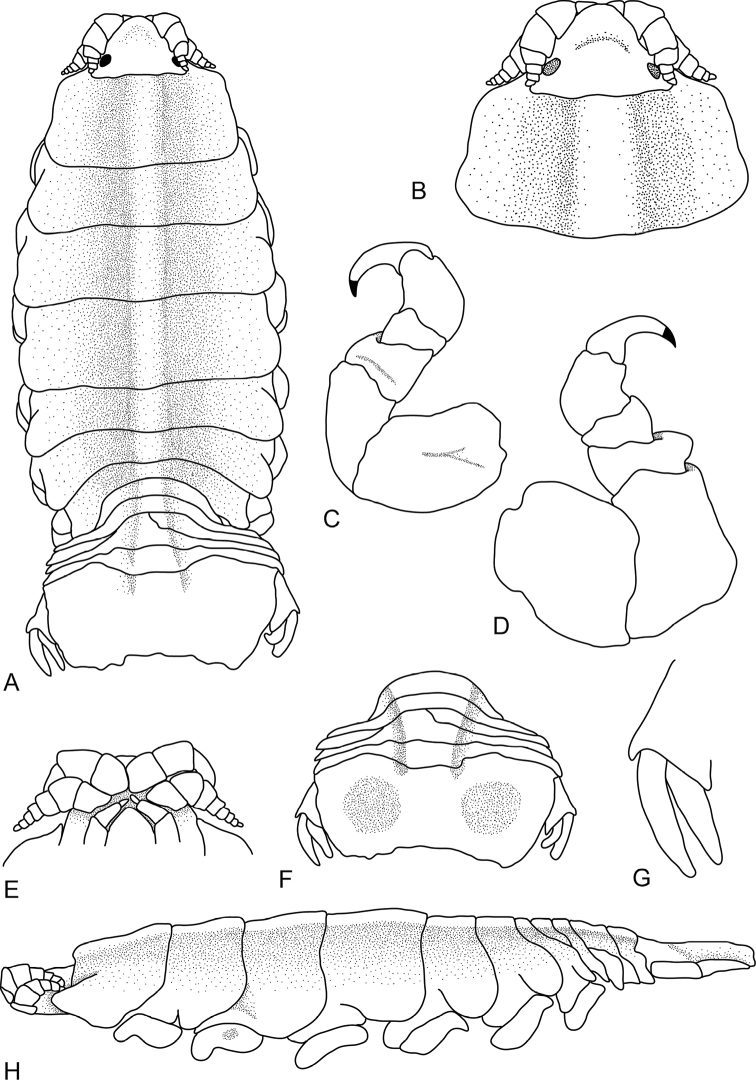
*Ceratothoa carinata* (Bianconi, 1869), female neotype (33 mm) (SAMC-A085795). **A** dorsal view **B** dorsal view of pereonite 1 and cephalon **C** pereonite 1 **D** pereopod 7 **E** ventral view of cephalon **F** dorsal view of pleotelson **G** uropod **H** lateral view.

##### Size.

Ovigerous female: 28.5–38 mm TL (10–14 mm W); non-ovigerous female: 13–34 mm TL; male: 10–12 mm TL (Bianconi 1869, Schioedte and Meinert 1883, Stebbing 1910, Trilles 1972b, c).

##### Distribution.

Western Indian Ocean and southwest Pacific Ocean: Mozambique (Bianconi 1869, Hilgendorf 1879, Schioedte and Meinert 1883); Great Redangs, Malay Peninsula (Lanchester 1902); Seychelles (Stebbing 1910); New Caledonia (Trilles 1972b, c, Bruce 2007); Red Sea (Trilles 2008); Japan (Nunomura 2006, Saito 2009, Nagasawa et al. 2014); and Australia (Martin et al. 2013).

##### Hosts.

On *Lutjanus adetii* (previously *Lutjanus amabilis*) (Trilles 1972b, c); *Pseudocaranx dentex* (see Nunomura 2006); *Decapterus muroadsi* (see Nunomura 2006, Saito 2009, Nagasawa et al. 2014); and *Selar crumenophthalmus* (see Martin et al. 2013, present study).

##### Remarks.

*Ceratothoa carinata* can be identified by the characteristic medial ridge extending longitudinally along the dorsal pereon surface. Furthermore, it has a laterally depressed and wider than long pleotelson; pereonite 7 with an enlarged carinate ischium and large bulbous protrusion on the merus; uropods reaching the distal edge of the pleotelson; as well as a concave posterior margin on the pleotelson.

Bianconi (1869) originally described this species from a single ovigerous female from Mozambique and stated that it was most similar to *Ceratothoa gaudichaudii* and *Ceratothoa trigonocephala*. Since then, another species, *Ceratothoa trillesi* (Avdeev 1979) was also described from the Australia–New Zealand region, and shared many morphological characteristics. There has been some confusion surrounding the synonymy of *Ceratothoa trillesi* with *Ceratothoa carinata* (see Avdeev 1979, Trilles 1994), however, these species differ substantially with *Ceratothoa trillesi* lacking the distinctive medial ridge, enlarged basis and ischium on pereopod 7, and a wide and depressed pleotelson seen in *Ceratothoa carinata*.

Species and names within *Ceratothoa* have been moved in and out of synonymy, an indication of both the difficulty of identifying and characterising species. Furthermore, many species are variable (Hadfield et al. 2014a) and species morphological boundaries are often unclear. In addition, the Cymothoidae also have groups of cryptic species such as has been seen in *Mothocya* (Bruce 1986) and *Anilocra* (Bunkley Williams and Williams 1981, Bruce 1987) and therefore the designation of a neotype is necessary in the long-term interests of nomenclatural stability.

Schioedte and Meinert (1883) mention a specimen from the type locality (Mozambique) that was originally deposited into Zoologisches Museum, Museum für Naturkunde, Homboldt-Universität, Berlin, Germany. Enquiries to that museum, as well as Muséum National d’Histoire Naturelle, Natural History Museum, London, the Naturalis Biodiversity Center and the Zoological Museum, University of Copenhagen, failed to locate any material that could be identified as the type for *Ceratothoa carinata*. It is highly probable that this specimen was destroyed in World War II or lost during relocation of the material. As cymothoid isopods are among the most misunderstood and difficult isopods to identify (Brusca 1981), a complete description (or redescription) of the type material is essential for accurate identifications and research on the species. The current material of *Ceratothoa carinata* was obtained from the type locality and corresponds with the original drawings of the species (Bianconi 1869). Both specimens have the noticeable medial ridge or hump running longitudinally down the length of the pereon. The pleotelson is medially concave and is wider than long. Furthermore, the uropods do not extend past the end of the pleotelson and the posterior margin of the pleotelson is indented medially; the eyes are small but clearly visible; and the antennae are stout and extend to the anterior margin of pereonite 1.

As the current specimen is undoubtedly *Ceratothoa carinata*, it is hereby designated as the neotype for the species, fulfilling all of the requirements necessary in the International Code of Zoological Nomenclature (Anon 1999, ICZN, Article 75).

#### Ceratothoa collaris

Taxon classificationAnimaliaIsopodaCymothoidae

Schioedte & Meinert, 1883

Figure 4

Cymothoa oestroïdes.—Lucas 1849: 78, pl. 8, figs 4a–c (see also page notes in Trilles 1972a p 1201). [not Ceratothoa oestroides (Risso, 1826)].
Ceratothoa collaris Schioedte & Meinert, 1883: 366–368, tab. XVI (Cym. XXIII) figs 8–9.—Carus 1885: 443.—Rokicki 1984a: 73; 1984b: 44–60, figs 9–12; 1985: 95–119, tabs. 1–3, fig. 8.—Trilles 1986: 623, tab. 1; 1994: 117.—Horton 2000: 1046–1047, figs 6a–c.—Ramdane and Trilles 2008: 173–178.—Bariche and Trilles 2008: 85–93, figs 1–5.
Meinertia collaris forma typica .—Monod 1924a: 31–34; 1924b: 430–432.—Trilles and Raibaut 1973: 277–278.—Capapé and Pantoustier 1976: 203.
Meinertia collaris forma africana .—Monod 1924a: 31–34; 1924b: 430–432; 1925: 103–104.—Trilles 1977: 10.
Meinertia collaris forma globuligera .—Monod 1924a: 31–34; 1924b: 430–432.
Meinertia collaris .—Trilles 1972b: 1240–1241, pl. I (1–2).—Dollfus and Trilles 1976: 822.—Moreira and Sadowsky 1978: 100, 110, 113–114, 120, 134.
Ceratothoa collaris forma africana .—Trilles 1979: 515, 522.
Ceratothoa collaris forma typica .—Trilles 1979: 521.

##### Material examined.

*Holotype*. National Museum of Natural History, Paris (MNHN-Is386) – ovigerous female specimen (40 mm TL) collected in Algeria by Lucas (Schioedte and Meinert 1883), host unknown, registered as *Meinertia collaris*, J.P. Trilles det. 17.12.1971 (n°40) (Trilles 1972b). Also noted: both right antennae are missing and some appendages are broken.

##### Description.

*Holotype female*. Length 40 mm, width 18 mm.

*Body* oval, 1.8 times as long as greatest width, dorsal surfaces slightly bumpy, widest at pereonite 4 and pereonite 5, most narrow at pereonite 1, lateral margins posteriorly ovate. *Cephalon* 0.6 times longer than wide, visible from dorsal view, triangular. *Frontal margin* rounded to form blunt rostrum. *Eyes* oval with distinct margins. *Antennula* more stout than antenna, shorter than antenna, with 7 articles. *Antenna* with 8 articles.

*Pereonite 1* with slight indentations, anterior border straight, anterolateral angle with distinct anterior projection, extend to middle of the eye. Posterior margins of pereonites smooth and slightly curved laterally. Coxae 2–3 narrow; with posteroventral angles rounded; 4–7 acute, posteriorly pointed; not extending past pereonite margin. Pereonites 1–4 increasing in length and width; 5–7 decreasing in length and width; becoming more progressively rounded posteriorly. *Pleon* with pleonite 1 most narrow, visible in dorsal view; pleonites posterior margin smooth, mostly concave. Pleonite 2 not overlapped by pereonite 7; posterolateral angles of pleonite 2 narrowly rounded. Pleonites 3–5 similar in form to pleonite 2; pleonite 5 free, not overlapped by lateral margins of pleonite 4, posterior margin produced medially. *Pleotelson* 0.4 times as long as anterior width, dorsal surface with medial furrow, lateral margins weakly convex, posterior margin damaged and shallowly emarginate.

*Pereopod 1* basis 1.7 times as long as greatest width; ischium 0.6 times as long as basis; merus proximal margin with bulbous protrusion; carpus with rounded proximal margin; propodus 1.6 times as long as wide; dactylus slender, 0.9 times as long as propodus, 2.1 times as long as basal width. *Pereopod 7* basis 1.4 times as long as greatest width; ischium 0.8 times as long as basis, without protrusions; merus proximal margin with large bulbous protrusion, merus 0.4 times as long as wide, 0.3 times as long as ischium; carpus 0.4 times as long as wide, 0.2 times as long as ischium, without bulbous protrusion; propodus 0.9 times as long as wide, 0.4 times as long as ischium; dactylus slender, 1.5 times as long as propodus, 2.3 times as long as basal width.

*Uropod* more than half the length of pleotelson, peduncle 0.9 times longer than rami, peduncle lateral margin without setae; rami not extending beyond pleotelson, marginal setae absent, apices narrowly rounded. *Endopod* apically rounded, 3.6 times as long as greatest width. *Exopod* extending to end of endopod, 4 times as long as greatest width, apically rounded.

**Figure 4.**
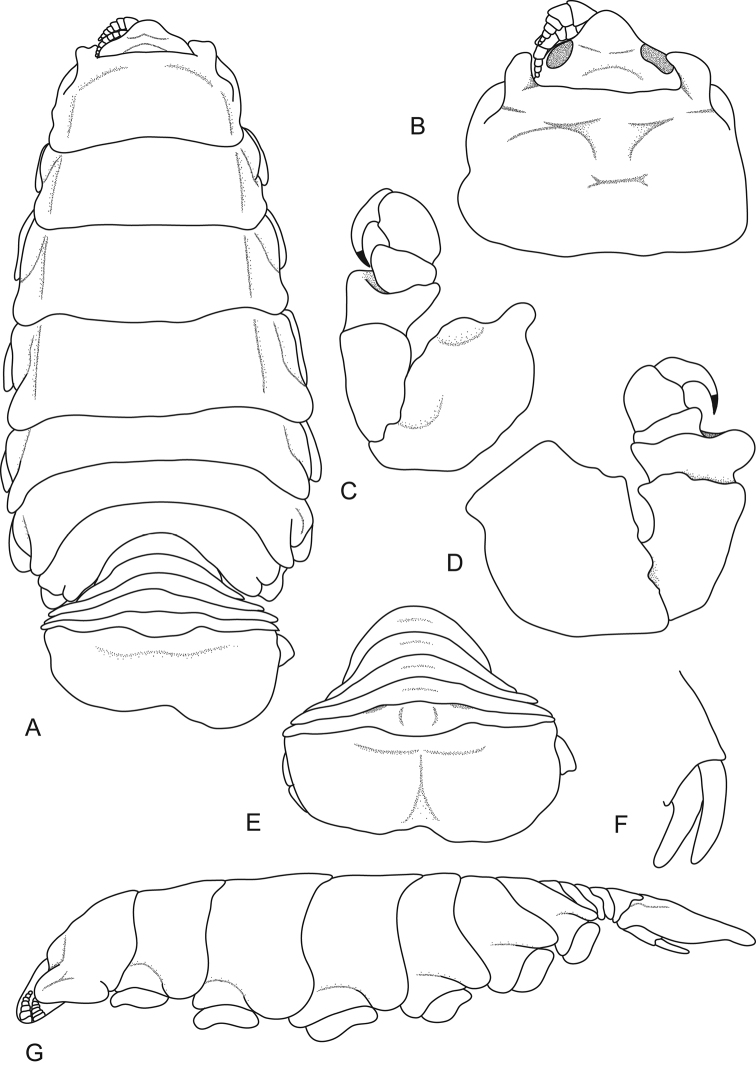
*Ceratothoa collaris* Schioedte & Meinert, 1883, female holotype (40 mm) (MNHN-Is386). **A** dorsal view **B** dorsal view of pereonite 1 and cephalon **C** pereopod 1 **D** pereopod 7 **E** dorsal view of pleotelson **F** uropod **G** lateral view.

##### Size.

Female: 18–40 mm TL (9–18 mm W); male: 9–22 mm TL (4–10 mm W); second pullus: 2.7–2.8 mm TL (0.7–0.8 mm W) (Monod 1924b, Capapé and Pantoustier 1976, Dollfus and Trilles 1976, Trilles 1977, 1979, Rokicki 1984a, b, Bariche and Trilles 2008).

##### Distribution.

Mediterranean and eastern Atlantic Ocean: Algeria (Schioedte and Meinert 1883, Lucas 1849, Trilles 1972b, 1979, Ramdane and Trilles 2008); Morocco (Monod 1924a, b, Trilles 1972b, Dollfus and Trilles 1976); Mauritania (Monod 1924a, b, Trilles 1972b, 1977, Dollfus and Trilles 1976); Tunisia (Trilles and Raibaut 1973, Capapé and Pantoustier 1976); Senegal (Trilles 1979); and Lebanon (Bariche and Trilles 2008).

*Ceratothoa collaris* is common in Tunisia (Trilles and Raibaut 1973) and Mauritania (Monod 1924a, Trilles 1977). This species has not been collected from the north or north-western Mediterranean countries despite many recent studies there. It has been found in southern areas of the Mediterranean, but never from Libya, Egypt, or Israel.

##### Hosts.

Frequently in the mouth of sparids from the genera *Dentex* and *Pagellus* (especially *Dentex gibbosus* and *Pagellus erythrinus*): *Dentex gibbosus* (previously *Dentex filosus*) (see Monod 1924a, b, Trilles 1972b, Trilles and Raibaut 1973, Rokicki 1984b), in *Pagellus erythrinus* (see Monod 1925, Bariche and Trilles 2008); mouth of *Pagellus acarne* (see Trilles 1972b, Dollfus and Trilles 1972); buccal cavity of *Dentex dentex*, *Dentex maroccanus*, *Spicara* sp., *Smaris* sp. and on ventral disc of *Raja miraletus* (see Trilles and Raibaut 1973); on *Sargus sargus*, *Pagellus bogaraveo*, pharynx of *Pagellus erythrinus*, and in gill cavity of a sparid (see Dollfus and Trilles 1972); on gill slits of *Torpedo marmorata* (see Capapé and Pantoustier 1976); in the mouth of *Pseudotolithus moorii* (previously *Corvina camaronensis*) (see Trilles 1977); in the mouth of *Smaris vulgaris* and on gills of *Pagellus* sp. (see Trilles 1979); *Dentex macrophthalmus*, *Pagrus pagrus* (see Rokicki 1984b); in the branchial cavity of *Pagrus caeruleostictus* (see Ramdane and Trilles 2008, Bariche and Trilles 2008); less frequent on *Dentex macrophthalmus*, *Pagellus acarne*, *Pagrus* sp., and rarely on *Dicentrarchus labrax* and *Epinephelus aeneus* (see Bariche and Trilles 2008).

Lucas (1849) considered *Ceratothoa collaris* to have a low host specificity (euryxenic) but Bariche and Trilles (2008) showed that there is a clear preference for Sparidae fish, particularly *Pagellus erythrinus*, which is commonly parasitised in Lebanon and Africa (Morroco and Algeria). Monod (1925) also stated how most of these isopods recorded from *Dentex filosus* were actually removed from *Pagellus erythrinus*, especially in the case of Ceratothoa collaris forma africana.

##### Remarks.

*Ceratothoa collaris* can be distinguished by the prominent anterolateral projections which do not extend past the eyes and form a collar-like structure from where it gets its name. It also has a wide pleotelson (same width or wider than pleon), uropods that do not extend past the pleotelson and a large bulbous protrusion on the pereopod 7 merus.

*Ceratothoa collaris* was described from Algeria, originally misidentified as *Ceratothoa oestroides* by Lucas (1849). Later, Monod (1924a) described three different forms of this species, namely Ceratothoa collaris forma globuligera, Ceratothoa collaris forma africana and Ceratothoa collaris forma typica based on morphological differences of their cephlon and antennae (Monod 1924a). Over the years, many researchers have identified other species where many forms are common, such as *Ceratothoa steindachneri* (see Horton 2000), but naming the different forms are not necessary, thus Bariche and Trilles (2008) removed the three *Ceratothoa collaris* forms.

#### Ceratothoa gilberti

Taxon classificationAnimaliaIsopodaCymothoidae

(Richardson, 1904)

Figure 5

Meinertia gilberti Richardson, 1904: 53, figs 32–33; 1905: 241–242, figs 247–249.—Schultz 1969: 157–158, fig. 237.
Codonophilus gilberti .—Nierstrasz 1931: 132.—Brusca 1977: 130; 1980: 230, 232, fig. 12.17.
Meinertia sp.—MacGinitie 1937: 1031, 1035, pl. I, fig. 1.
Ceratothoa gilberti .—Wallerstein 1980: 232.—Brusca 1981: 178–182, figs 21a–d, figs 22a–l.—Avdeev 1982a: 65–67; 1982b: 69–77.—Brusca and Iverson 1985: 49.—Trilles 1994: 119.—Espinosa-Pérez and Hendrickx 2001: 48.

##### Material examined.

*Lectotype* [here designated]. United States National Museum, USA (USNM 1254761) – female (22 mm TL; 9.5 mm W) collected from Mazatlan, Sinaloa (Mexico) in mouth of *Mugil hospes* (see Richardson 1904). The left side of pereonites 3–5 were damaged. *Paralectotypes*. Two males (11–12 mm TL; 4–5 mm W), same data as lectotype (USNM 29080).

##### Description.

*Lectotype female*. Length 22 mm, width 9.5 mm.

*Body* oval, 1.8 times as long as greatest width, dorsal surfaces slightly bumpy, widest at pereonite 4 and pereonite 5, most narrow at pereonite 1, lateral margins posteriorly ovate. *Cephalon* 0.6 times longer than wide, visible from dorsal view, triangular. *Frontal margin* rounded to form blunt rostrum. *Eyes* oval with distinct margins. *Antennula* more stout than antenna, shorter than antenna, with 7 articles. *Antenna* with 8 articles.

*Pereonite 1* with slight indentations, anterior border straight, anterolateral angle with distinct anterior projection, extend to middle of the eye. Posterior margins of pereonites smooth and slightly curved laterally. Coxae 2–3 narrow; with posteroventral angles rounded; 4–7 acute, posteriorly pointed; not extending past pereonite margin. Pereonites 1–4 increasing in length and width; 5–7 decreasing in length and width; becoming more progressively rounded posteriorly. *Pleon* with pleonite 1 most narrow, visible in dorsal view; pleonites posterior margin smooth, mostly concave. Pleonite 2 not overlapped by pereonite 7; posterolateral angles of pleonite 2 narrowly rounded. Pleonites 3–5 similar in form to pleonite 2; pleonite 5 free, not overlapped by lateral margins of pleonite 4, posterior margin produced medially. *Pleotelson* 0.4 times as long as anterior width, dorsal surface with medial furrow, lateral margins weakly convex, posterior margin damaged and shallowly emarginate.

*Pereopod 1* basis 1.7 times as long as greatest width; ischium 0.6 times as long as basis; merus proximal margin with bulbous protrusion; carpus with rounded proximal margin; propodus 1.6 times as long as wide; dactylus moderately slender, 0.9 times as long as propodus, 2.1 times as long as basal width. *Pereopod 7* basis 1.4 times as long as greatest width; ischium 0.8 times as long as basis, without protrusions; merus proximal margin with large bulbous protrusion, merus 0.4 times as long as wide, 0.3 times as long as ischium; carpus 0.4 times as long as wide, 0.2 times as long as ischium, without bulbous protrusion; propodus 0.9 times as long as wide, 0.4 times as long as ischium; dactylus slender, 1.5 times as long as propodus, 2.3 times as long as basal width.

*Uropod* more than half the length of pleotelson, peduncle 0.9 times longer than rami, peduncle lateral margin without setae; rami not extending beyond pleotelson, marginal setae absent, apices narrowly rounded. *Endopod* apically rounded, 3.6 times as long as greatest width. *Exopod* extending to end of endopod, 4 times as long as greatest width, apically rounded.

**Figure 5.**
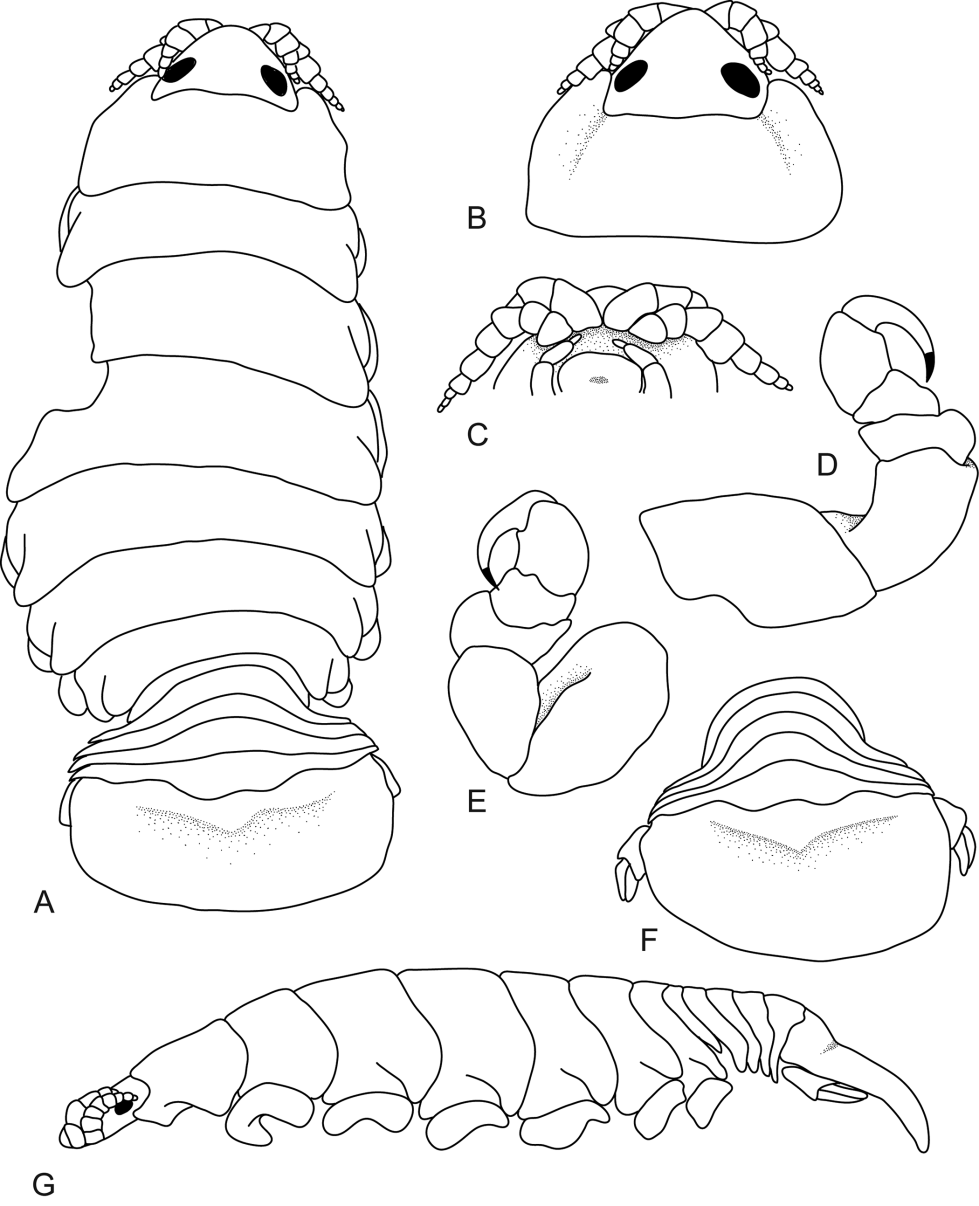
*Ceratothoa gilberti* (Richardson, 1904), female lectotype (22 mm) (USNM 1254761). **A** dorsal view **B** dorsal view of pereonite 1 and cephalon **C** ventral view of cephalon **D** pereopod 1 **E** pereopod 7 **F** dorsal view of pleotelson **G** lateral view.

##### Size.

Female: 16–29 mm TL (8–14 mm W) (Brusca 1981).

##### Distribution.

Known from the south-western coast of northern America in the Gulf of California region: from southern California, USA (MacGinitie 1937, Brusca 1981, Espinosa-Pérez and Hendrickx 2001) and the west coast of Baja California to Puerto Penasco and Mazatlan, Mexico (Richardson 1904, Nierstrasz 1931, Brusca 1977, Brusca 1981, Espinosa-Pérez and Hendrickx 2001).

##### Hosts.

On tongue of the mullet *Mugil cephalus* (see MacGinitie 1937, Brusca 1977, 1981); from the mullet, *Mugil hospes* (see Richardson 1904, Brusca 1977, 1981); and from a “flatfish” (Brusca 1981).

##### Remarks.

*Ceratothoa gilberti* has an elongate body; pleon as wide as pereon; short uropods; a elongate, triangular cephalon; short anterolateral projections on pereonite 1; and a large pleotelson with a rounded posterior margin. Furthermore, Brusca (1981) previously noted that *Ceratothoa gilberti* lacks an appendix masculina in the male. The female specimen is here designated as the lectotype and redescribed.

*Ceratothoa gilberti* has been infrequently collected and seems to be confined to the region around the Gulf of California. It has only been found on mullet species and has often been compared to *Ceratothoa gaudichaudii* (which has recently been placed into *species inquirenda* by Martin et al. [2015]).

#### Ceratothoa gobii

Taxon classificationAnimaliaIsopodaCymothoidae

Schioedte & Meinert, 1883

Figure 6

Ceratothoa Gobii Schioedte & Meinert, 1883: 356–358, tab. XV (Cym. XXII) figs 12–13.
Ceratothoa gobii .—Carus 1885: 443.—Trilles 1994: 119.—Horton 2000: 1042–1043.
Meinertia gobii .—Montalenti 1948: 36.

##### Material examined.

*Holotype*. Museum of Comparative Zoology, USA (MCZ 3707) – female (12 mm TL; 5 mm W), from the sand goby, *Gobius minutus*, from Messina, Italy, coll. Haeckel. Specimen with broken antennae and damaged pereonite 2.

##### Description.

*Holotype female*. Length 12 mm, width 5 mm.

*Body* elongate, 1.9 times as long as greatest width, dorsal surfaces smooth and polished in appearance, widest at pereonite 5, most narrow at pereonite 1, lateral margins slightly convex. *Cephalon* 0.7 times longer than wide, visible from dorsal view, triangular. *Frontal margin* rounded to form blunt rostrum. *Eyes* oval with distinct margins, one eye 0.3 times width of cephalon; 0.6 times length of cephalon.

*Pereonite 1* smooth, anterior border straight, anterolateral angle with small distinct produced point and produced past frontal margin of cephalon, extend to middle of the eye. Posterior margins of pereonites smooth and straight. With posteroventral angles rounded; coxae 4–7 rounded; not extending past pereonite margin. Pereonites 1–5 increasing in length and width; 6–7 decreasing in length and width; becoming more progressively rounded posteriorly. *Pleon* with pleonite 1 most narrow, visible in dorsal view; pleonites posterior margin smooth, mostly concave. Pleonite 2 not overlapped by pereonite 7; posterolateral angles of pleonite 2 narrowly rounded. Pleonites 3–5 similar in form to pleonite 2; pleonite 5 free, not overlapped by lateral margins of pleonite 4, posterior margin produced medially. *Pleotelson* 0.4 times as long as anterior width, dorsal surface smooth, lateral margins weakly convex, posterior margin subtruncate and shallowly emarginate.

*Pereopod 1* basis 1.4 times as long as greatest width; ischium 0.7 times as long as basis; merus proximal margin with bulbous protrusion; carpus with rounded proximal margin; propodus 1.7 times as long as wide; dactylus slender, as long as propodus, 3 times as long as basal width. *Pereopod 7* basis as long as greatest width; ischium 0.8 times as long as basis, without protrusions; merus proximal margin with large bulbous protrusion, merus 0.4 times as long as wide, 0.3 times as long as ischium; carpus 0.7 times as long as wide, 0.3 times as long as ischium, with slight bulbous protrusion; propodus 1.1 times as long as wide, 0.4 times as long as ischium; dactylus slender, 1.6 times as long as propodus, 2.7 times as long as basal width.

*Uropod* same length or slightly longer than the pleotelson, peduncle 0.6 times longer than rami, peduncle lateral margin without setae; rami extending beyond pleotelson, marginal setae absent, apices narrowly rounded. *Exopod* extending to end of endopod.

**Figure 6.**
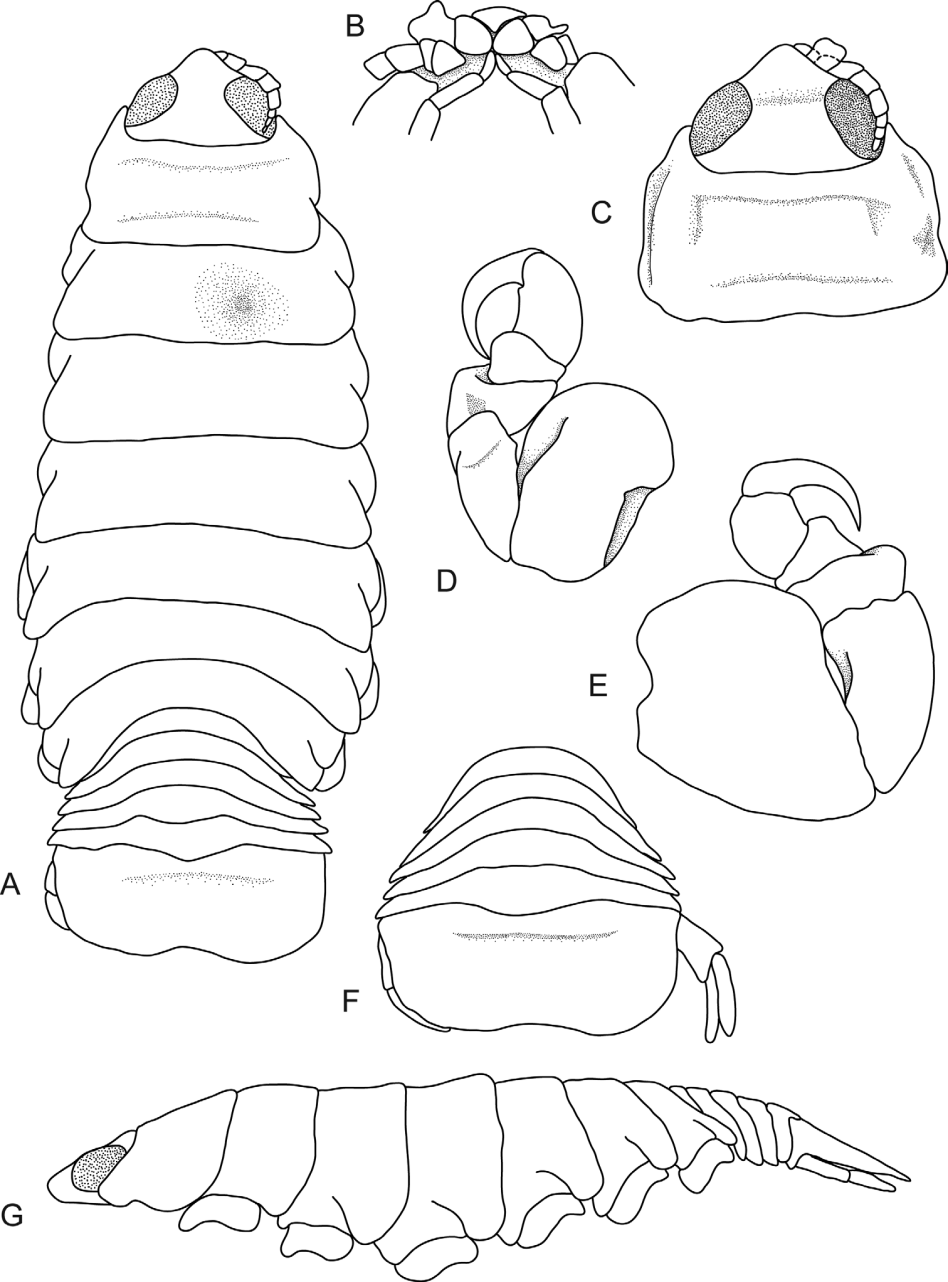
*Ceratothoa gobii* Schioedte & Meinert, 1883, female holotype (12 mm) (MCZ 3707). **A** dorsal view **B** ventral view of cephalon **C** dorsal view of pereonite 1 and cephalon **D** pereopod 1 **E** pereopod 7 **F** dorsal view of pleotelson **G** lateral view.

##### Remarks.

*Ceratothoa gobii* has a triangular cephalon with a sub-truncate rostrum; large eyes which together take up more than half of the cephalon; uropods which extend past the posterior margin of the pleotelson; short anterolateral projections on pereonite 1; and pleonites 1–5 gradually becoming wider.

This species is based on the description of a single specimen by Schioedte and Meinert (1883) without mention of a type host; however, *Gobius minutus* (now *Pomatoschistus minutus*) is listed on the information in the museum bottle. This species has only been collected once.

#### Ceratothoa guttata

Taxon classificationAnimaliaIsopodaCymothoidae

(Richardson, 1910)

Figure 7

Meinertia guttata Richardson, 1910: 20–21, fig. 19.
Codonophilus guttatus .—Nierstrasz 1931: 132.
Meinertia venusta Avdeev, 1978: 30–32, pl. 1.
Ceratothoa venusta .—Avdeev 1981: 1160–1167, fig. 3; 1990: 32–42, figs 1–6.—Trilles 1986: 625, tab. 1; 1994: 129–130.
Ceratothoa guttata .—Bruce and Bowman 1989: 4–7, figs 3–4.—Trilles 1994: 119–120.—Kensley 2001: 232.—Bruce, Lew Ton and Poore 2002: 172.—Martin, Bruce and Nowak 2015a: 271–272.

##### Material examined.

*Lectotype* [here designated]. United States National Museum, USA (USNM 1254762) – female (17 mm TL; 7 mm W), 7 Feb 1908, obtained off Jolo Island, Philippines; from a flying fish 4–5 inches long (Richardson 1910). Specimen’s left side slightly damaged from pereonite 4 to 6. *Paralectotypes*. Five females (13.5–16 mm TL; 5–6 mm W), same data as lectotype (USNM 40914).

##### Description.

*Lectotype female*. Length 17 mm, width 7 mm.

*Body* oval and elongate, twice as long as greatest width, dorsal surfaces smooth and polished in appearance, widest at pereonite 5, most narrow at pereonite 1, lateral margins ovate. *Cephalon* 0.6 times longer than wide, visible from dorsal view, triangular. *Frontal margin* rounded to form blunt rostrum. *Eyes* irregular in outline. *Antennula* and antenna stout and same length. Antennula with 7 articles, *antenna* with 8 articles.

*Pereonite 1* smooth, anterior border straight, anterolateral angle with small distinct anterior projection extending to base of eyes. Posterior margins of pereonites slightly produced medially. Coxae 2–3 wide, with posteroventral angles rounded; 4–7 large and produced on pereonite margins, not extending past pereonite margin. Pereonites 1–5 increasing in length and width; 6–7 decreasing in length and width; 2–5 subequal. *Pleon* with pleonite 1 most narrow, visible in dorsal view; pleonites posterior margin smooth, mostly concave. Pleonite 2 not overlapped by pereonite 7; posterolateral angles of pleonite 2 forming acute point. Pleonites 3–5 similar in form to pleonite 2; pleonite 5 free, not overlapped by lateral margins of pleonite 4, posterior margin produced medially. *Pleotelson* 0.6 times as long as anterior width, dorsal surface smooth, lateral margins weakly convex, posterior margin shallowly emarginate.

*Pereopod 1* basis 1.5 times as long as greatest width; ischium 0.8 times as long as basis; merus proximal margin with large bulbous protrusion; carpus with straight proximal margin; propodus as long as wide; dactylus slender, 1.4 times as long as propodus, 2.4 times as long as basal width. *Pereopod 7* basis 0.8 times as long as greatest width; ischium 1.3 times as long as basis, with a large proximal bulbous protrusion overlapping merus; merus proximal margin with large distal bulbous protrusion, merus 0.7 times as long as wide, 0.3 times as long as ischium; carpus 0.7 times as long as wide, 0.3 times as long as ischium, without bulbous protrusion; propodus 1.4 times as long as wide, 0.5 times as long as ischium; dactylus slender, 1.4 times as long as propodus, 2.6 times as long as basal width.

*Uropod* more than half the length of pleotelson, peduncle 0.8 times longer than rami.

**Figure 7.**
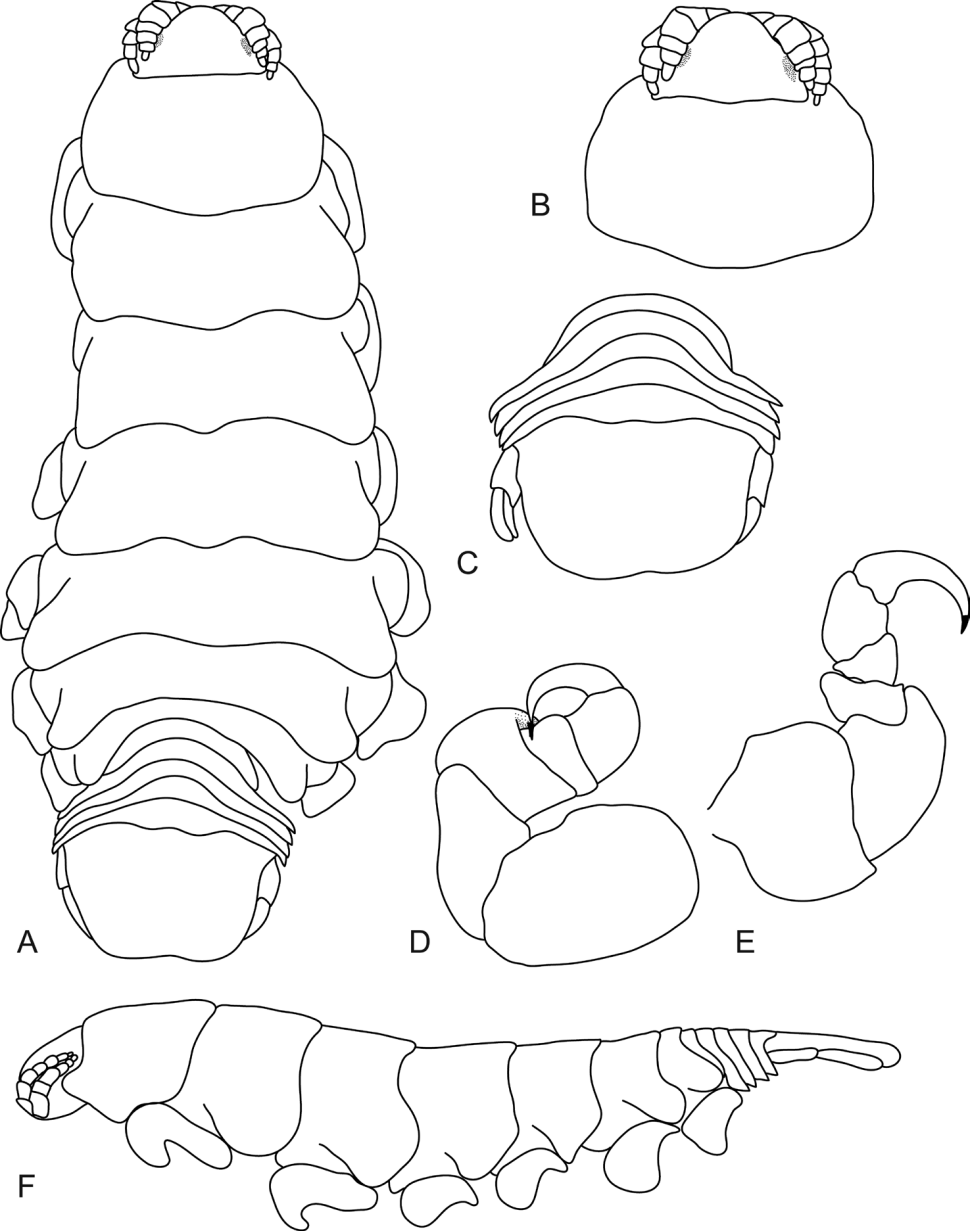
*Ceratothoa guttata* (Richardson, 1910), female lectotype (17 mm) (USNM 1254762). **A** dorsal view **B** dorsal view of pereonite 1 and cephalon **C** dorsal view of pleotelson **D** pereopod 1 **E** pereopod 7 **F** lateral view.

##### Other material

. Holotype of *Ceratothoa venusta*. Russian Pacific Federal Fisheries Research Institute (AGK 75054) – on flying fish, *Parexocoetus brachypterus*, from the Red Sea (Avdeev 1978).

##### Size.

Ovigerous females: 14.5–23.0 mm TL; non-ovigerous females: 15.5–16.5 mm TL; males: 5.4–7.4 mm TL (Bruce and Bowman 1989).

##### Distribution.

Central and Western Indo-Pacific: Philippines (Richardson 1910, Nierstrasz 1931, Bruce and Bowman 1989, Kensley 2001); Red Sea (Avdeev 1978, Bruce and Bowman 1989, Kensley 2001); Madagascar; Australia; and Taiwan (Bruce and Bowman 1989, Kensley 2001).

##### Hosts.

In mouths of flying fish, *Parexocoetus brachypterus* (see Bruce and Bowman 1989, Avdeev 1978).

##### Remarks.

*Ceratothoa guttata* is distinguished by the elongate body widest at pereonite 5; uropods which do not extend past the posterior margin of the pleotelson; a narrow pleon; an expanded merus on pereopod 1; and an expanded ischium and merus on pereopod 7.

*Ceratothoa guttata* is considered to be highly host specific as it has, to date, only been reported from a single host, *Parexocoetus brachypterus*. Avdeev (1978) briefly described *Meinertia venusta* from the Red Sea on *Parexocoetus brachypterus*, comparing it to *Ceratothoa guttata*. Bruce and Bowman (1989) revised *Ceratothoa guttata* and synonymised *Ceratothoa venusta* with *Ceratothoa guttata* after comparing drawings of the two species. Similar morphology of pereopods 1 and 7, coxae, the pleon and the pereon, as well as the host specificity all confirm this synonymy. The largest female is here redescribed and reillustrated and designated as lectotype.

#### Ceratothoa italica

Taxon classificationAnimaliaIsopodaCymothoidae

Schioedte & Meinert, 1883

Figure 8

Ceratothoa italica Schioedte & Meinert, 1883: 347–350, tab. XV (Cym. XXII), figs 1–4.—Carus 1885: 442.—Trilles 1979: 521; 1986: 624, tab. 1; 1994: 121; 2008: 23.—Rokicki 1984b: 129–131, fig. 32; 1985: 95–119, tab. 1–3, fig. 6.—Trilles, Radujković and Romestand 1989: 289, fig. 8.—Horton 2000: 1047, figs 7c–e.—Öktener and Trilles 2004: 145–154.—Ramdane, Bensouilah and Trilles 2007: 67–74.
Meinertia italica .—Monod 1924a: 34; 1924b: 432–434.—Montalenti 1948: 42–46, figs 11–14, tab. 5, pl. 3.—Trilles 1964b: 106; 1972a: 1212–1215, figs 156–187, pl. II, figs 10–12; 1972b: 1238–1240.—Dollfus and Trilles 1976: 823.
Ceratothoa italica Identity uncertain: Ceratothoa italica.—Ateş, Trilles, İşmen and Yiğin 2006: 375–380.—Trilles 2008: 23.

##### Material examined.

*Lectotype* [here designated]. Zoological Museum, University of Copenhagen (ZMUC CRU-6914) – female specimen (36 mm TL, 17 mm W) collected in Rijeka, Croatia (previously called Fiume), Adriatic Sea by Budde-Lund (Schioedte and Meinert 1883), host unknown. Also noted: the female has a broken pereonite 1, pleonite 2 and antenna; pereopod 1 missing and other pereopods are damaged and missing dactylii. *Paralectotypes*. Thirty-seven pullus stage (4–5 mm TL), same data as lectotype (ZMUC CRU-8669); Eighty-two pullus stage (4–5 mm TL), same data as lectotype, label reads “Stor female udt. Som pectotype” (ZMUC CRU-9124).

##### Description.

*Lectotype female*. Length 36 mm, width 17 mm.

*Body* rectangular and elongate, 1.7 times as long as greatest width, dorsal surfaces smooth and polished in appearance, widest at pereonite 5 and pereonite 6, most narrow at pereonite 1, lateral margins subparallel. *Cephalon* 0.5 times longer than wide, visible from dorsal view, triangular. *Eyes* irregular in outline, one eye 0.2 times width of cephalon; 0.2 times length of cephalon.

*Pereonite 1* with unique bulbous orientation, anterior border anteriorly produced medially, anterolateral angle wide, with inwardly produced point, extend to anterior margin of eyes. Posterior margins of pereonites slightly produced medially. Coxae 4–7 rounded, not extending past pereonite margin. Pereonites 1–4 increasing in length and width; 5–7 decreasing in length and width; 6 and 7 narrower and becoming more progressively rounded posteriorly. *Pleon* with pleonite 1 same width as other pleonites (except pleonite 5), visible in dorsal view; pleonites posterior margin smooth, mostly concave. Posterolateral angles of pleonite 2 narrowly rounded. Pleonites 3–5 similar in form to pleonite 2; pleonite 5 free, not overlapped by lateral margins of pleonite 4, posterior margin with 2 indented points. *Pleotelson* 0.5 times as long as anterior width, dorsal surface with medial furrow, lateral margins weakly convex, posterior margin subtruncate.

*Pereopod 1* basis 1.5 times as long as greatest width; ischium 0.9 times as long as basis; merus proximal margin with large bulbous protrusion; carpus with straight proximal margin; propodus 1.1 times as long as wide; dactylus slender, 1.2 times as long as propodus, 2.3 times as long as basal width. *Pereopod 7* basis 1.2 times as long as greatest width; ischium 0.8 times as long as basis, without protrusions; merus proximal margin with slight bulbous protrusion, merus 0.5 times as long as wide, 0.4 times as long as ischium; carpus 0.5 times as long as wide, 0.3 times as long as ischium, without bulbous protrusion; propodus 1.1 times as long as wide, 0.5 times as long as ischium; dactylus slender, 1.4 times as long as propodus, 2.5 times as long as basal width.

*Uropod* same length as pleotelson, peduncle 0.5 times longer than rami, peduncle lateral margin without setae; rami extending to pleotelson apex, marginal setae absent, apices narrowly rounded.

**Figure 8.**
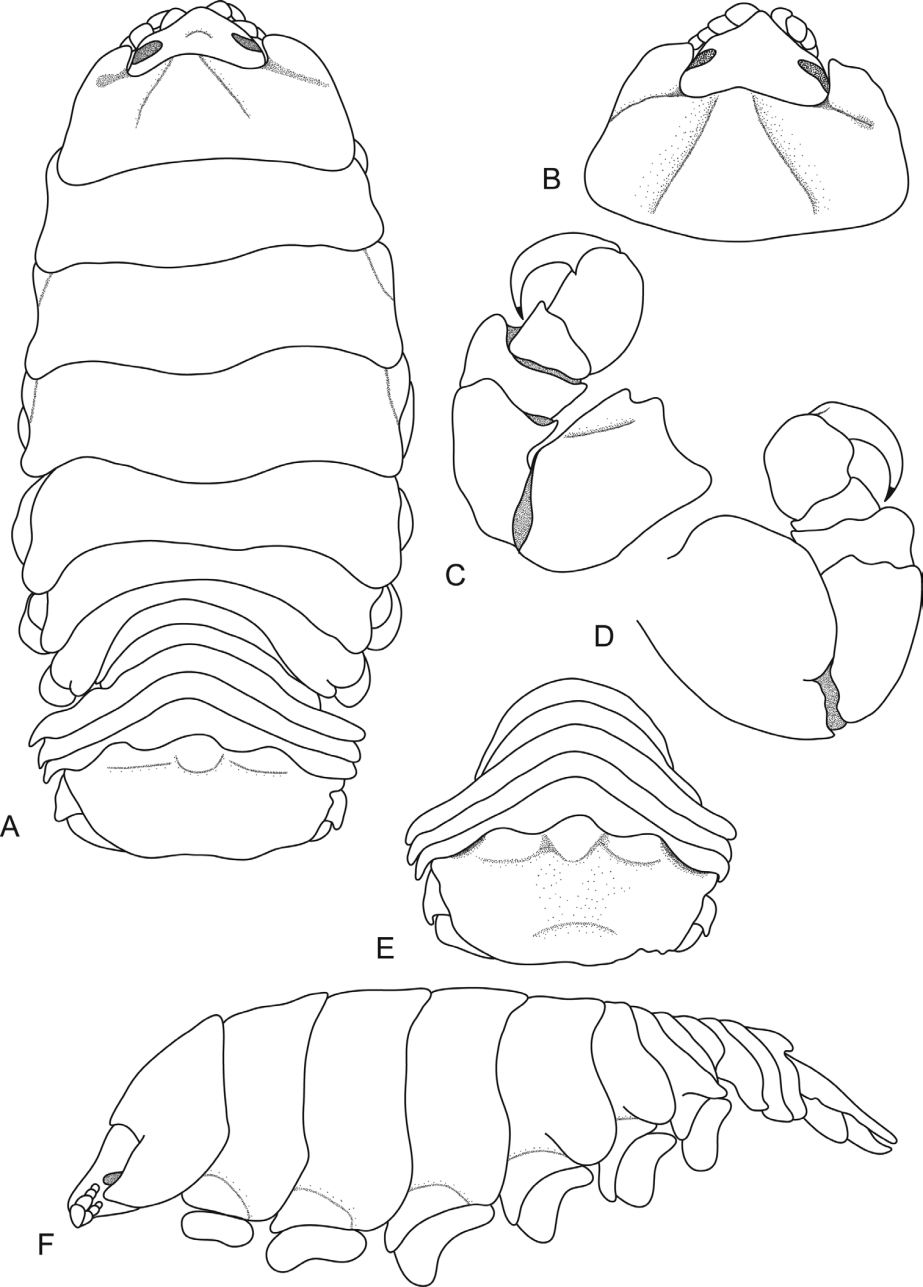
*Ceratothoa italica* Schioedte & Meinert, 1883, female lectotype (36 mm) (ZMUC CRU-6914). **A** dorsal view **B** dorsal view of pereonite 1 and cephalon **C** pereopod 1 **D** pereopod 7 **E** dorsal view of pleotelson **F** lateral view.

##### Size.

Ovigerous female: 15–30 mm TL; male: 8–15 mm TL; second pullus: 3 mm TL (Montalenti 1948, Trilles 1972a, Rokicki 1984b).

##### Distribution.

Mediterranean region and north-western Africa: Adriatic Sea (Schioedte and Meinert 1883, Carus 1885, Öktener and Trilles 2004); Mauritania (Monod 1924a, 1924b, Trilles 1972b); Italy (Montalenti 1948); Galite Islands, Tunisia, France, Morocco (Trilles 1972b); Montenegro (Trilles et al. 1989); Aegean Sea (Ateş et al. 2006); and Algeria (Ramdane et al. 2007).

##### Hosts.

In mouth of *Spondyliosoma cantharus* (previously *Cantharus lineatus*) (Monod 1924b, Trilles 1972b); mouth of *Lithognathus mormyrus* (previously *Pagellus mormyrus*) and other bream (Montalenti 1948, Trilles 1964b); on *Pagellus erythrinus* (see Trilles 1964b); on *Oblada melanura*; on Mustèle; in mouth of *Sargus* (see Trilles 1972b); occurs in Sparidae fishes (Rokicki 1985); in the mouth of *Diplodus sargus* (see Trilles et al. 1989); in the buccal cavity of *Diplodus annularis* (see Ramdane et al. 2007).

##### Remarks.

*Ceratothoa italica* can be distinguished by the arched body; large bulbous protrusion on the merus of pereopod 1; a pointed rostrum; and uropods that do not extend past the pleotelson. This species also has a prominent projection in the middle of pereonite 1 (hump-like) and a pleon which is usually as wide as the pereon.

Ateş et al. (2006) stated that cymothoid isopods identified as *Ceratothoa italica* were collected from the eggs of *Nephrops norvegicus*. As cymothoids are fish parasites, this interaction is an unusual association and is most likely accidental. Similarly, the record of *Ceratothoa italica* (originally labelled as a *Cymothoa* sp. [SMF-3515]) from Senckenberg Research Institute, Frankfurt, Germany (Trilles 2008) seems doubtful as the specimen was collected from Norway and this species has only previously been recorded from the Mediterranean Sea region.

The female ZMUC CRU-6914 is here designated as the lectotype and the pullus stages in the same bottle and the other sample are paralectotypes (ZMUC CRU-8869, 9124).

#### Ceratothoa oestroides

Taxon classificationAnimaliaIsopodaCymothoidae

(Risso, 1826)

Figure 9

**Figure 9.**
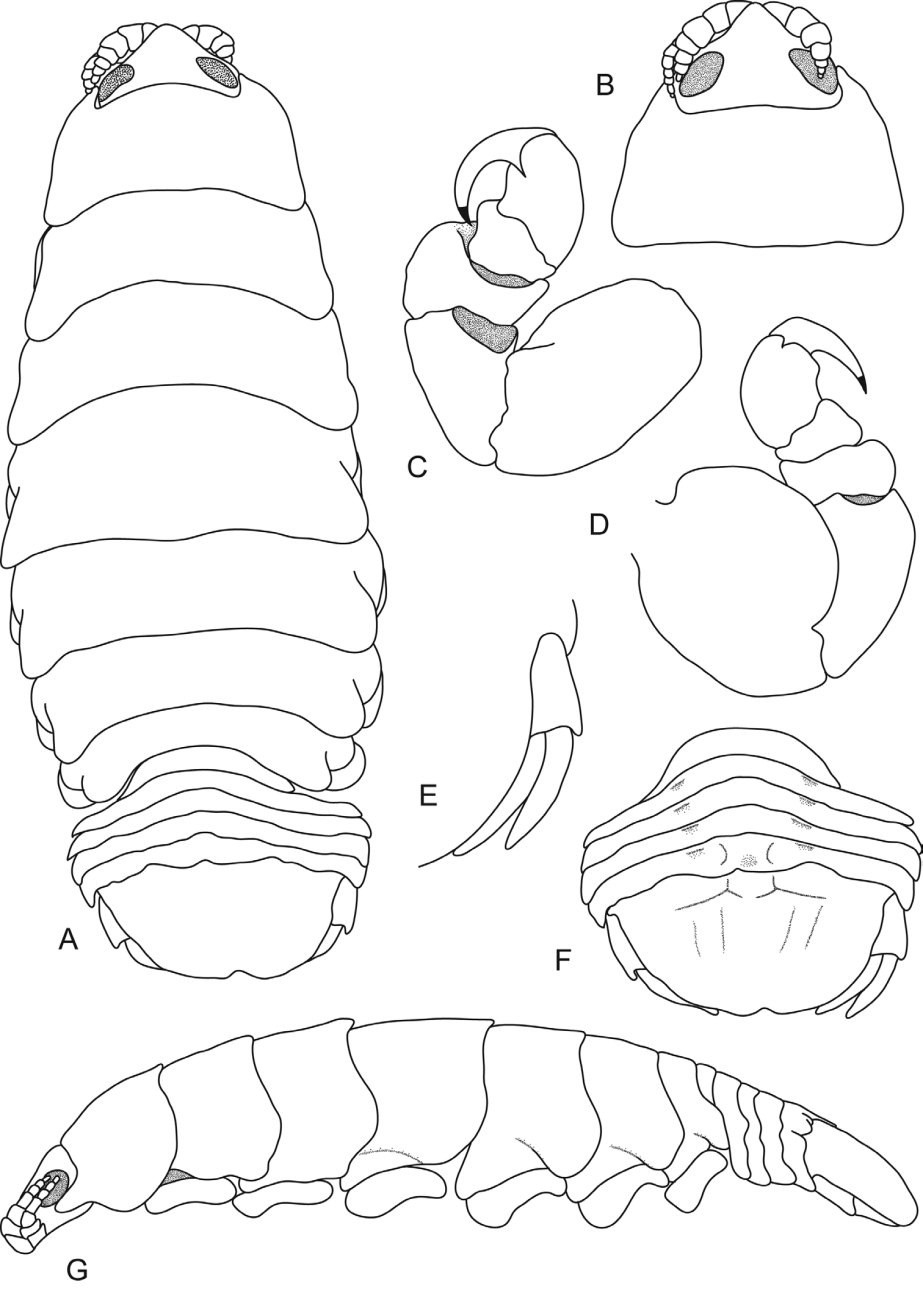
*Ceratothoa oestroides* (Risso, 1826), female lectotype (22 mm) (MNHN-IU-2014-17478). **A** dorsal view **B** dorsal view of pereonite 1 and cephalon **C** pereopod 1 **D** pereopod 7 **E** uropod **F** dorsal view of pleotelson **G** lateral view.

**Figure 10.**
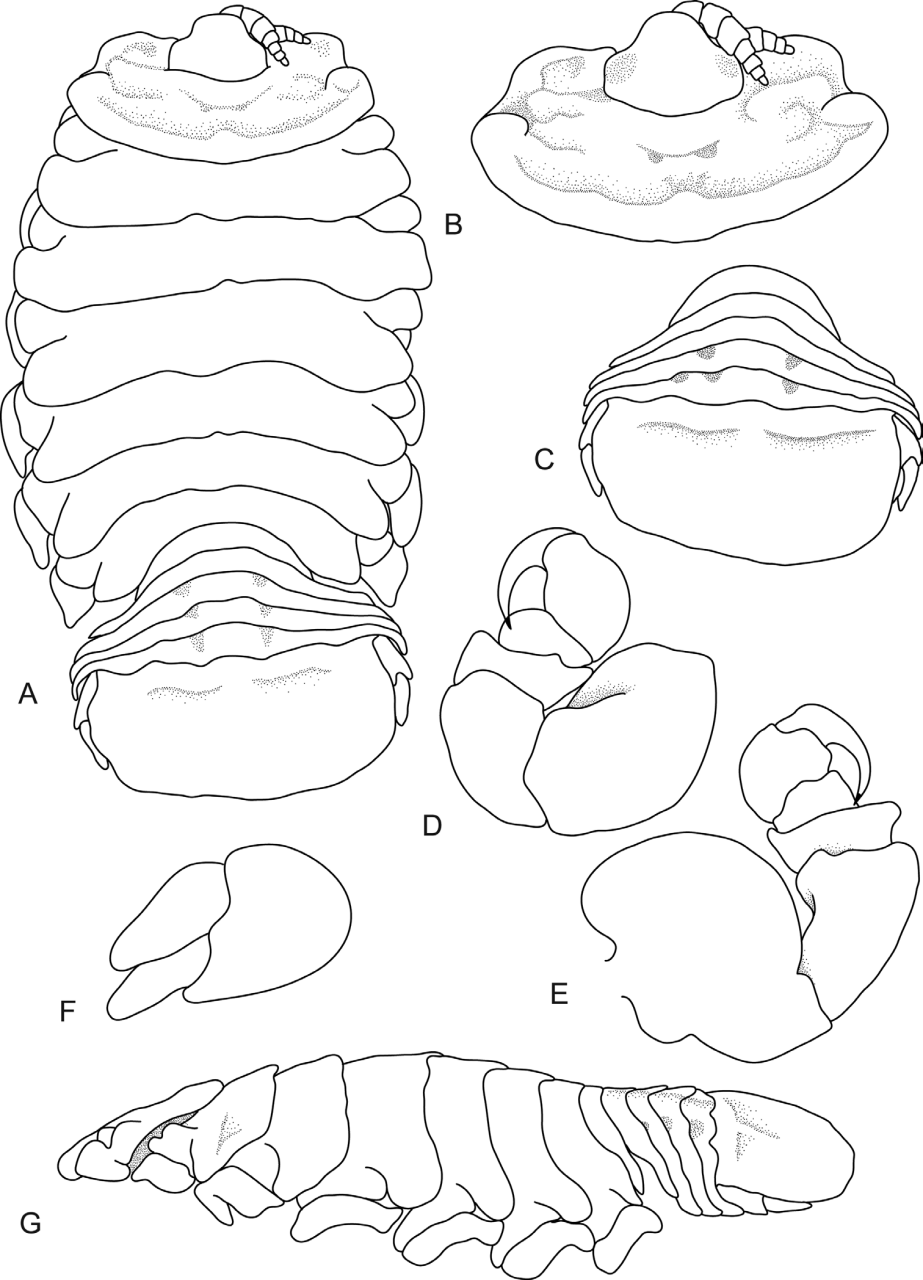
*Ceratothoa verrucosa* (Schioedte & Meinert, 1883), female lectotype (40 mm) (RMNH.CRUS.I.7706). **A** dorsal view **B** dorsal view of pereonite 1 and cephalon **C** dorsal view of pleotelson **D** pereopod 1 **E** pereopod 7 **F** uropod **G** lateral view.

**Figure 11.**
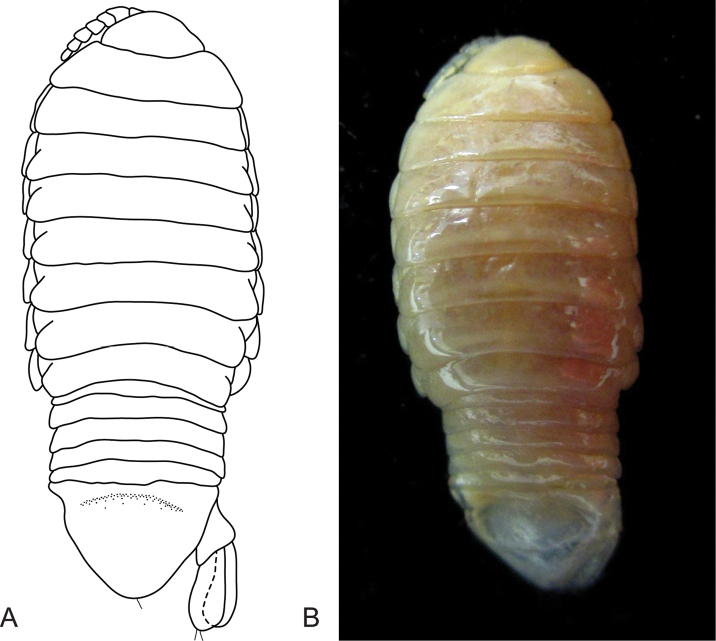
*Ceratothoa transversa* (Richardson, 1901), immature male holotype (17 mm) (USNM 9728). **A, B** dorsal view.

Canolira œstroïdes Risso, 1826: 123.
Cymothoa oestroides .—Milne Edwards 1840: 272.—White 1847: 110.—Lucas 1849: 78, pl. 8 (fig. 4).—Hope 1851: 33.—Heller 1866: 737–738.—Barcelo Combis 1875: 68.—Stalio 1877: 236.—Stossich 1880: 45.—Bonnier 1887: 133–134.—Monticelli 1890: 420–421.—Gerstaecker 1901: 181, 256–257.—Sanada 1941: 209.
Cymothoa (Meinertia) oestroides .—Taschenberg 1879: 245.—Dollfus 1922: 287.
Ceratothoa oestroides .—Schioedte and Meinert 1883: 350–356, tab. XV (Cym. XXII) figs 5–11.—Carus 1885: 442.—Barrois 1888: 12.—Gourret 1891: 14–15, pl. 4, figs 10–11.—Bolivar 1892: 133.—Koelbel 1892: 107, 115.—Norman 1905: 13.—Dudich 1931: 18.—Trilles 1979: 515, 521; 1986: 624, tab. 1; 1994: 122–124; 2008: 23.—Renaud, Romestand and Trilles 1980: 467–476.—Brusca 1981: 178.—Romestand, Thuet and Trilles 1982: 79–89.—Rokicki 1984b: 116–119, fig. 28; 1985: 95–119, tab. 1–3, fig. 7.—Radujković, Romestand and Trilles 1984: 164–166, figs 2–3; 1985: 106.—Trilles, Radujković and Romestand 1989: 289–291, fig. 9.—Šarušić 1999: 110–112.—Charfi-Cheikhrouha, Zghidi, Ould Yarba and Trilles 2000: 143–150, tab. 4.—Horton 2000: 1047–1048, fig. 7a–b.—Horton and Okamura 2001: 181–187.—Rodríguez-Sánchez, Serna and Junoy 2001: 154.—Mladineo 2002: 97–102; 2003: 97–101; 2006: 438–442.—Mladineo and Valic 2002: 304–310.—Junoy and Castello 2003: 307.—Korun and Akayli 2004: 123–132.—Öktener and Trilles 2004: 145–154.—Ramdane, Bensouilah and Trilles 2007: 67–74.—Pérez-Del Olmo, Fernández, Gibson, Raga and Kostadinova 2007: 152, 154.—Solak, Öktener, Trilles and Solak 2007: 237–238.—Oguz and Öktener 2007: 79–83.—Matašin and Vučinić 2008: 363–367.—Ramdane and Trilles 2008: 173–178.—Kirkim, Kocataş, Katağan and Sezgin 2008: 382–385.—Gökpinar, Özgen and Yildiz 2009: 188–190.—Mladineo, Šegvić and Grubišić 2009: 160–167.—Innal and Kirkim 2012: A13–A16.
Ceratothoa sargorum Gourret, 1891: 16, pl. 1, fig. 17; pl. 5, figs 1–4.
Meinertia oestroides .—Thielemann, 1910: 36.—Nierstrasz 1915: 8.—Monod 1923a: 82–83; 1923b: 18–19; 1924a: 34; 1924b: 432.—Montalenti 1948: 47–50, figs 15–17, tab. 6, pl. 4.—Amar 1951: 530.—Balcells 1953: 548–552.—Euzet and Trilles 1961: 190–192.—Trilles 1962: 118–123; 1964b: 107; 1972a: 1201–1208, figs 90–136, pl. 1 figs 6–9, pl. 3 fig 20; 1972b: 1233–1235 (part); 1977: 8–9.—Vu–Tân–Tûe 1963: 225–232.—Berner 1969: 93–94.—Roman 1970: 163–208; 1979: 501–514.—Trilles and Raibaut 1971: 73–75, photos 2–3; 1973: 274.—Thampy and John 1974: 575, 580–582.—Romestand 1974: 571–591; 1979: 423–448, pl. 1–4.—Romestand and Trilles 1976a: 87–92, fig. 1; 1976b: 663–665; 1977a: 91–95, figs 1–2; 1977b: 47–53, figs 1–11; 1979: 195–202.—Romestand, Voss–Foucart, Jeuniaux and Trilles 1976: 981–988.—Dollfus and Trilles 1976: 822.—Chaigneau 1977: 403–418.—Romestand, Janicot and Trilles 1977: 171–172, 178–179, pl. 3, figs 10–14.—Quignard and Zaouali 1980: 357.—Thuet and Romestand 1981: 15–33.—Brusca 1981: 125.—Radujković 1982: 153–161.—Wägele 1987: 1–398.
Cymothea oestroides [sic].—Odon de Buen 1916: 363.
Ceratothoa (Meinertia) oestroides .—Brusca 1981: 123.
Ceratothoa oestroides Not Cymothoa oestroides.—Gibert i Olivé 1920: 88.
Ceratothoa oestroides Excluded (identity uncertain): Meinertia oestroides.—Trilles 1972b: 1233–1235 (part).
Ceratothoa oestroides .—Trilles 1981: 587.

##### Material examined.

*Lectotype* [here designated]. National Museum of Natural History, Paris (MNHN-IU-2014-17478) – female specimen (22 mm TL; 8 mm W), collected from the Mediterranean Sea; J.P. Trilles checked 17.12.1971, host unknown (n°6) (Trilles 1972b). *Paralectotype*. Female specimen (21 mm TL; 8 mm W), same data as holotype (Trilles 1972b) (MNHN-IU-2007-4240). Also noted: the two females were in the same bottle as a female *Cymothoa parallela* (19 mm TL; 6 mm W) (MNHN-IU-2014-17479).

##### Description.

*Lectotype female*. Length 22 mm, width 8 mm.

*Body* oval and elongate, 1.9 times as long as greatest width, dorsal surfaces smooth and polished in appearance, widest at pereonite 4 and pereonite 5, most narrow at pereonite 1, lateral margins posteriorly ovate. *Cephalon* 0.6 times longer than wide, visible from dorsal view, triangular. *Frontal margin* rounded to form blunt rostrum. *Eyes* oval with distinct margins, one eye 0.3 times width of cephalon; 0.4 times length of cephalon. *Antennula* more stout than antenna, comprised of 7 articles. *Antenna* comprised of 8 articles.

*Pereonite 1* smooth, anterior border straight, anterolateral angle with small distinct produced point, extend to middle of the eye. Posterior margins of pereonites smooth and slightly curved laterally. Coxae 2–3 narrow, with posteroventral angles rounded; 4–7 rounded, not extending past pereonite margin. Pereonites 1–4 increasing in length and width; 5–7 decreasing in length and width; 6 and 7 narrower. *Pleon* with pleonite 1 most narrow and same width as other pleonites, visible in dorsal view; pleonites posterior margin smooth, mostly concave. Pleonite 2 not overlapped by pereonite 7; posterolateral angles of pleonite 2 narrowly rounded. Pleonites 3–5 similar in form to pleonite 2; pleonite 5 free, not overlapped by lateral margins of pleonite 4, posterior margin with 2 indented points or produced medially. *Pleotelson* 0.5 times as long as anterior width, dorsal surface with lateral indent, lateral margins weakly convex, posterior margin rounded with medial indent.

*Pereopod 1* basis 1.7 times as long as greatest width; ischium 0.6 times as long as basis; merus proximal margin with large bulbous protrusion; carpus with rounded proximal margin; propodus 1.5 times as long as wide; dactylus slender, 0.9 times as long as propodus, 2.3 times as long as basal width. *Pereopod 7* basis 1.1 times as long as greatest width; ischium 0.9 times as long as basis, with slight bulbous protrusion; merus proximal margin with large bulbous protrusion, merus 0.6 times as long as wide, 0.3 times as long as ischium; carpus 0.7 times as long as wide, 0.3 times as long as ischium, without bulbous protrusion; propodus 1.4 times as long as wide, 0.6 times as long as ischium; dactylus slender, 0.9 times as long as propodus, 2.4 times as long as basal width.

*Uropod* same length or slightly longer than the pleotelson, peduncle 0.8 times longer than rami, peduncle lateral margin without setae; rami extending to pleotelson apex, marginal setae absent, apices narrowly rounded.

##### Size.

Ovigerous female: 12–30 mm; non-ovigerous female: 11–24.5 mm TL; male: 3.5–13 mm TL; second stage pullus: 3.3–4 mm TL; first stage pullus: 3.1 mm TL (Schioedte and Meinert 1883, Montalenti 1948, Trilles 1972a, 1977, 1979, Rokicki 1984b, Radujković et al. 1985).

##### Distribution.

Throughout the Mediterranean and eastern Atlantic: especially France and Algeria (Risso 1826, Milne Edwards 1840, Schioedte and Meinert 1883, Thielemann 1910, Trilles 1964b); Adriatic (Heller 1866, Stalio 1877); Aegean Sea (Horton and Okamura 2001, Trilles 2008); Straits of Gibraltar and the Alborán Sea (Rodríguez-Sánchez et al. 2001); Turkey (Öktener and Trilles 2004, Solak et al. 2007, Innal and Kirkim 2012); and the eastern Atlantic islands and north-west Africa (Barrois 1888, Koelbel 1892, Monod 1924b, Trilles 1972b, 1979, Dollfus and Trilles 1976).

##### Hosts.

Common in the mouth and branchial regions of the bogue, *Boops boops* (see Schioedte and Meinert 1883, Gourret 1891, Monod 1923b, Balcells 1953, Vu–Tân–Tûe 1963, Berner 1969, Trilles and Raibaut 1971, 1973, Romestand and Trilles 1976a, 1977a, b, 1979, Roman 1979, Taschenberg 1879, Renaud et al. 1980, Radujković 1982, Trilles et al. 1989, Charfi-Cheikhrouha et al. 2000, Pérez-Del Olmo et al. 2007, Ramdane et al. 2007, Matašin and Vučinić 2008, Ramdane and Trilles 2008, Kirkim et al. 2008); “rare parasite of wrasses (Labres)” (Bonnier 1887); in the mouth of *Diplodus sargus* (see Gourret 1891); in buccal cavity of *Spicara maena* (see Gourret 1891, Trilles 1962, Berner 1969, Roman 1979, Radujković 1982, Charfi-Cheikhrouha et al. 2000, Kirkim et al. 2008); on the gills of two *Phycis phycis* (recorded as *Phycis mediterraneas*) (see Koelbel 1892); from the mouth of *Trachurus trachurus* (see Dollfus 1922, Trilles and Raibaut 1971, 1973, Dollfus and Trilles 1976, Charfi-Cheikhrouha et al. 2000, Ramdane et al. 2007); in the mouth of *Spicara* and “*Box*” sp. (see Montalenti 1948, Amar 1951); in the buccal cavity of *Diplodus vulgaris* (see Monod 1923b, Amar 1951); buccal and gill cavity of *Diplodus annularis* (see Monod 1923b, Trilles and Raibaut 1971, 1973, Trilles 1972b, Dollfus and Trilles 1976, Radujković 1982, Trilles et al. 1989, Charfi-Cheikhrouha et al. 2000); in the mouth of red mullet, *Mullus barbatus* (see Trilles 1962, Roman 1970); on sardine, *Sardina pilchardus* (see Trilles 1962, 1979, Charfi-Cheikhrouha et al. 2000); buccal cavity of *Spicara* sp. and gill cavity of *Uranoscopus scaber* (see Trilles and Raibaut 1973); in mouth cavity of *Pagellus erythrinus* (see Romestand and Trilles 1979, Radujković et al. 1985, Trilles et al. 1989); in mouth of *Spicara melanurus* (previously *Smaris melanurus*), *Sargus bellottii*, and *Abudefduf saxatilis* (see Trilles 1979); *Trachurus mediterraneus* (see Radujković 1982, Trilles et al. 1989); picarels, *Spicara smaris* (see Trilles et al. 1989, Ramdane et al. 2007, Matašin and Vučinić 2008, Ramdane and Trilles 2008); *Pagellus acarne* (see Ramdane et al. 2007, Ramdane and Trilles 2008); *Dicentrarchus labrax* (see Šarušić 1999, Horton and Okamura 2001, Mladineo 2002, 2003); *Sparus aurata* (see Šarušić 1999, Horton and Okamura 2001, Mladineo 2003); on the tongue of *Scorpaena notata*, *Liza aurata* and *Scorpaena porcus* (see Charfi-Cheikhrouha et al. 2000); in the black seabream, *Spondyliosoma cantharus* (see Charfi-Cheikhrouha et al. 2000, Gökpinar et al. 2009); from *Rostroraja alba* and *Zeus faber* (see Kirkim et al. 2008).

##### Remarks.

*Ceratothoa oestroides* can be distinguished by having an acute rostrum; short antennae; prominent eyes; uropods which extend to or past the posterior pleotelson margin; large protrusion on the merus of pereonite 1; and a large carina on pereopod 7 in female specimens, as well as the appendix masculina absent in male specimens.

*Ceratothoa sargorum* Gourret, 1891, found on *Sargus rondeletii*, was described from a single female with large eggs, almost a millimetre in diameter (Gourret 1891). This species was later synonymised with *Ceratothoa oestroides* as seen in Radujković et al. (1984). The original drawings by Gourret (1891) resemble the syntypic material of *Ceratothoa oestroides* and confirm this synonymy.

There have been reported cases of *Ceratothoa oestroides* involved in hyperparasitism. Dollfus (1922) recorded an unusual association with *Ceratothoa oestroides* and a monogenean, *Allodiclidophora charcoti* (Dollfus, 1922) (Diclidophoridae) after being collected from *Ceratothoa oestroides* in the mouth of *Trachurus trachurus* from Oviedo. Similarly, Monod (1923b) stated that the ectoparasite was found on one *Ceratothoa oestroides* specimen from the mouth of *Box vulgaris*.

*Ceratothoa oestroides* has often been misidentified as *Ceratothoa oxyrrhynchaena*. Both species use similar host fish and have an overlapping distribution range, but they are distinguished by the morphology of the seventh pair of pereopods in the female. It should be noted that male *Ceratothoa oestroides* does not possesses an appendix masculina. We regard the records of *Ceratothoa oestroides* from the Caribbean (Trilles 1972b, 1981) as unconfirmed, and are not included in the synonymy and distribution for the species.

Horton (2000) recently revised this species including full synonymy, host and distribution notes for *Ceratothoa oestroides* and listed the two female syntypes from MNHN (sample No. 6) as the type material. The type material had not previously been redescribed and no holotype had been designated by Risso (1826), so one female was hereby designated as a lectotype in order to provide a precise type-based description for the species.

#### Ceratothoa verrucosa

Taxon classificationAnimaliaIsopodaCymothoidae

(Schioedte & Meinert, 1883)

Figure 10

Oniscus Ceti Spengler, 1775: 312 [*nomen nudum*].
Rhexana verrucosa Schioedte & Meinert, 1883: 291–296, tab. XI (Cym. XVIII) figs 5–10.—Thielemann 1910: 34–35, tab. 3.—Sanada 1941: 209–217.—Bruce and Bowman 1989: 2.—Trilles 1994: 134–135.
Rhexanella verrucosa .—Stebbing 1911: 179.—Nierstrasz 1915: 87.—Shiino 1951: 83, figs 1a–b.—Trilles 1972b: 1255–1256, pl. II, figs 17–18.—Nunomura 1981: 52.—Avdeev 1982b: 69–77.—Bruce and Bowman 1989: 2.—Yamaguchi 1993: 193–194, fig. 21.
Ceratothoa verrucosa .—Yamauchi and Nunomura 2010: 73, figs 7–8.
Ceratothoa verrucosa Identity uncertain: Rhexanella verrucosa.—Nierstrasz 1931: 131.

##### Material examined.

*Lectotype*. National Museum of Natural History (Naturalis), Leiden, Netherlands (RMNH.CRUS.I.7706) – ovigerous female (40 mm TL; 21 mm W), collected from Japan, unknown host, 1823-1829, coll: Siebold, Ph.F.v (designated by Yamaguchi 1993). *Paralectotypes*. Immature female (26 mm TL; 10.5 mm W), two males (16–19 mm TL; 7–8 mm W), same data as holotype (RMNH.CRUS.I.39). Female slightly twisted, non-ovigerous, damaged pereopods and pleopods with uropods missing.

##### Description.

*Lectotype female*. Length 40 mm, width 21 mm.

*Body* oval, 1.9 times as long as greatest width, dorsal surfaces slightly bumpy, widest at pereonite 4, most narrow at pereonite 1, lateral margins slightly convex. *Cephalon* 0.7 times longer than wide, visible from dorsal view, subtriangular. *Frontal margin* rounded to form blunt rostrum. *Eyes* irregular in outline. *Pereonite 1* with unique bulbous orientation, anterior border slightly indented, anterolateral angle with large wide projections, extend to anterior margin of eyes. Posterior margins of pereonites slightly damaged and bumpy. Coxae 2–3 wide; 4–7 large and produced on pereonite margins, not extending past pereonite margin. Pereonites subequal. *Pleon* with pleonite 1 most narrow, visible in dorsal view; pleonites posterior margin not smooth, mostly concave. Pleonite 2 not overlapped by pereonite 7; posterolateral angles of pleonite 2 narrowly rounded. Pleonites 3–5 similar in form to pleonite 2; pleonite 5 free, not overlapped by lateral margins of pleonite 4, posterior margin produced medially. *Pleotelson* 0.5 times as long as anterior width, dorsal surface with lateral indent, lateral margins weakly convex, posterior margin evenly rounded. *Antennula* more stout than antenna, same length as antenna, consisting of 7 articles. *Antenna* consisting of 9 articles.

*Pereopod 1* basis 1.4 times as long as greatest width; ischium 0.7 times as long as basis; merus proximal margin with large bulbous protrusion; carpus with rounded proximal margin; propodus 1.2 times as long as wide; dactylus slender, 0.9 times as long as propodus, 1.9 times as long as basal width. *Pereopod 7* basis 0.8 times as long as greatest width; ischium as long as basis, with slight bulbous protrusion; merus proximal margin with large bulbous protrusion, merus 0.4 times as long as wide, 0.3 times as long as ischium; carpus 0.6 times as long as wide, 0.9 times as long as ischium, without bulbous protrusion; propodus 0.7 times as long as wide, 0.4 times as long as ischium; dactylus slender, 1.8 times as long as propodus, twice as long as basal width.

*Uropod* half the length of pleotelson, peduncle as long as rami, peduncle lateral margin without setae; rami not extending beyond pleotelson, marginal setae absent, apices narrowly rounded.

##### Size.

Ovigerous females: 27–50 mm TL (15.5–25.5 mm W); non-ovigerous females 20.5–40 mm TL; males 15–35 mm TL (11 mm W); larvae 3.5 mm TL (Schioedte and Meinert 1883, Thielemann 1910, Nierstrasz 1931, Trilles 1972b, Nunomura 1981, Yamauchi and Nunomura 2010).

##### Distribution.

Known from Japan and surrounding islands (Schioedte and Meinert 1883, Thielemann 1910, Sanada 1941, Trilles 1972b, Nunomura 1981, Yamauchi and Nunomura 2010).

##### Hosts.

In the mouth of *Pagrus major* (previously *Pagrosomus major*) (“Tai” in Japanese) (Sanada 1941, Shiino 1951, Nunomura 1981, Yamauchi and Nunomura 2010).

##### Remarks.

*Ceratothoa verrucosa* is distinguished by the large, oval body; wide anterolateral projections on pereonite 1; pleon as wide as pereon; and short uropods not extending to the posterior margin of the pleotelson.

This species was originally thought to infect a Greenland whale (Spengler 1775) but Spengler (1775) may have tried to relate this isopod to the whale lice *Cyamus ceti*. Cymothoid isopods had not been mentioned often before this time and researchers had some confusion with their identification. Schioedte and Meinert (1883), however, stated that this species (referred as “*Oniscus Ceti*”) was undoubtedly the same as their “*Rhexana*” species. There is no detailed description and no type material for Spengler’s (1775) species, so the original description is a *nomen nudum*; the correct authority for the species is Schioedte and Meinert (1883) who first made the name available. The name *Rhexana* was preoccupied, and the genus was then changed to *Rhexanella* Stebbing, 1911, this name being later synonymised with *Ceratothoa* (see Hadfield et al. 2014b).

Nierstrasz (1931) listed this species from the East Indian Archipelago, specifically from Nangamessi Bay, Sumba (Indonesia) after being collected during the H.M. *Siboga* expeditions. If confirmed, this distribution range would increase the distribution of *Ceratothoa verrucosa* (previously only found from Japan and only from one host, *Pagrus major*).

### Species excluded from *Ceratothoa*

#### Ceratothoa argus

Taxon classificationAnimaliaIsopodaCymothoidae

(Haswell, 1881) nomen dubium

Codonophilus argus Haswell, 1881: 471, pl. XVI, fig. 1; 1882: 283; 1885: 1001.—Stebbing 1893: 356.—Hale 1926: 223–226.—Barnard 1940: 404.—Trilles 1972c: 5, 7.

##### Holotype.

Deposition unknown.

##### Distribution.

Australia (Haswell 1881).

##### Hosts.

Under the bell of a *Rhizostoma* (see Haswell 1881).

##### Remarks.

This species was described from an immature specimen (4 mm in length) in only a few sentences and a single figure. It was found under the bell of a *Rhizostoma* in Port Jackson (Sydney, New South Wales) and noted as being similar to *Aegathoa* in many ways, but differed in the sudden narrowing of the body at the commencement of the pleon, and the uniramous character of the caudal appendages. Hale (1926) synonymised *Ceratothoa argus* with *Ceratothoa imbricata* as it appeared similar to the brood young of *Ceratothoa imbricata* and according to the label it was also reported as coming from the jelly blubber, *Catostylus mosaicus* (recorded as *Catostylus mosaicus*).

Due to the species being based on a single immature specimen (as well as a lack of a type specimen and an incomplete description), this species is hereby considered *nomen dubium*.

#### Ceratothoa poutassouiensis

Taxon classificationAnimaliaIsopodaCymothoidae

(Penso, 1939) nomen dubium

Meinertia poutassouiensis Brian, 1939: 20–24 [*nomen nudum*].
Meinertia (Ceratothoa) potassoniensis .—Penso 1939: 1, figs 1–2 [*lapsus*].
Ceratothoa poutassouiensis .—Trilles 1994: 127.—Horton 2000: 1042.

##### Hosts.

*Micromesistius poutassou* (previously *Gadus potassoa*).

##### Remarks.

These two species names, published in the same year, refer to the same species. Brian (1939) stated that “… *this isopod circa 2 cm in length … a species of* Meinertia *deserves to be described as it seems to be a new species, I hope to be able to publish the description of this species which I call* Meinertia poutassouiensis”. Horton (2000) added that both authors cited the species as found on *Micromesistius poutassou* (as *Gadus potassoa*), but it was inadequately described with two uninformative figures in Penso (1939). This lack of sufficient information, lack of types to redescribe the species and lack of the location of the type material lead Horton (2000) to place *Ceratothoa poutassouiensis* (Brian, 1939) into *nomen nudum*. The mention of size alone by Brian (1939) does not meet the criteria of availability, specifically ICZN Article 13.1.1, as it does not differentiate or define the species; Brian’s name is therefore not available so the authority has to be Penso (1939) as Penso provided two figures of the species thereby validating the name. The correct spelling of the epithet remains that proposed by Brian (1939). Given the lack of a descriptive data, lack of types and lack of a specific type locality, the species is here regarded as *nomen dubium*.

#### Ceratothoa transversa

Taxon classificationAnimaliaIsopodaCymothoidae

(Richardson, 1901) species inquirenda

Figure 11

Meinertia transversa Richardson, 1900: 221 [*nomen nudum*].
Meinertia transversa Richardson, 1901: 529–530, figs 12–13; 1905: 243, figs 250–252.—Menzies and Frankenberg 1966: 9.—Schultz 1969: 156, fig. 234.—Menzies and Kruczynski 1983: 39.
Ceratothoa transversa .—Brusca 1981: 178.—Trilles 1994: 128.

##### Material examined.

*Holotype*. United States National Museum, USA (USNM 9728) – immature specimen (17 mm TL; 7 mm W) collected from the Gulf of Mexico, *Albatross* Station 2395, U.S.F.C., 347 fms (635 metres), host unknown.

##### Distribution.

Between the Mississippi Delta and Cedar Keys, Florida (Richardson 1900, 1901, 1905, Menzies and Frankenberg 1966, Schultz 1969).

##### Hosts.

Unknown.

##### Remarks.

*Ceratothoa transversa* was originally noted as having a cephalon only slightly immersed in pereonite 1; long antennae extending past pereonite 1; uropods slightly longer than the pleotelson; and a sub-triangular pleotelson.

Richardson (1900) mentioned the name *Meinertia transversa* without differentiating characters or figures, without type locality, type deposition or type host, referring only to an "in press" paper and as such that name is a *nomen nudum*. Richardson (1901) later gave a short description of the species with figures of the cephalon and pleon were given. Schultz (1969) commented that this species is probably based on a young individual and Menzies and Kruczynski (1983) further stated that this species has never been adequately illustrated and probably also represents an *Aegathoa*-stage specimen as uropodal rami and pleotelson are shown with setae by Richardson (1900, 1901, 1905).

Examination of the holotype confirmed that it is an immature specimen. The antennae extend into the middle of pereonite 1, the pleon is almost as wide as the pereon, there are a few setae on the uropods and pleotelson, the pleopods overlap and the appendix masculina is absent. Without an adult female to characterise the species, and no known hosts to assist in directing the collection of a new specimen, the identity of this species is uncertain and therefore *Ceratothoa transversa* is hereby placed into *species inquirenda*.

#### Ceratothoa triglae

Taxon classificationAnimaliaIsopodaCymothoidae

Gourret, 1891 species inquirenda

Ceratothoa triglae Gourret, 1891: 19–20, pl. 11, figs 14–19.

##### Remarks.

*Ceratothoa triglae* (Gourret, 1891) was described from a male specimen measuring 7 mm TL. Gourret (1891) reported that it measured at least four times longer than wide and that it was found on the cheeks and belly of *Chelidonichthys lucerna* (previously *Trigla corax*). Second stage pulli were also found on the cheeks, probably newly released, along with the female; however, no other mention is made of the female after this statement. This species was subsequently placed into synonymy with *Cymothoa parallela* (see Radujković et al. 1984) and maintained there by Trilles (1994) and Horton (2000). As there is no known type material, and the description is based on a male specimen, this species can only be regarded as *species inquirenda*.

#### Elthusa parva

Taxon classificationAnimaliaIsopodaCymothoidae

(Richardson, 1910) comb. n.

Figure 12

Meinertia parva Richardson, 1910: 21, fig. 20.
Codonophilus parvus .—Nierstrasz 1931: 132.
Ceratothoa parva .—Trilles 1994: 127.

##### Material examined.

*Holotype*. United States National Museum, USA (USNM 40938) – female (19 mm TL; 8.5 mm W), collected from Opol, Mindanao, Philippines, 4 August 1909, host unknown (Richardson 1910).

##### Remarks.

*Ceratothoa parva* was originally described as having distinct eyes; rounded anterolateral processes on pereonite 1 which extend half the length of the cephalon; and short uropods which do not reach the end of the pleotelson.

Examination of the holotype revealed many characters not usually present in *Ceratothoa*. Pleonite 1 is as wide as the other pleonites (usually narrower), pleonite 4 is slightly wider than pleonite 5, slender and short antennae, and, most significantly, the bases of the antennae do not touch (a defining characteristic of *Ceratothoa*). These characters, together with the shape of the head and pereopod morphology all agree well with the generic characters for *Elthusa* Schioedte & Meinert, 1884 (see Bruce 1990), and the species is here placed in that genus.

**Figure 12.**
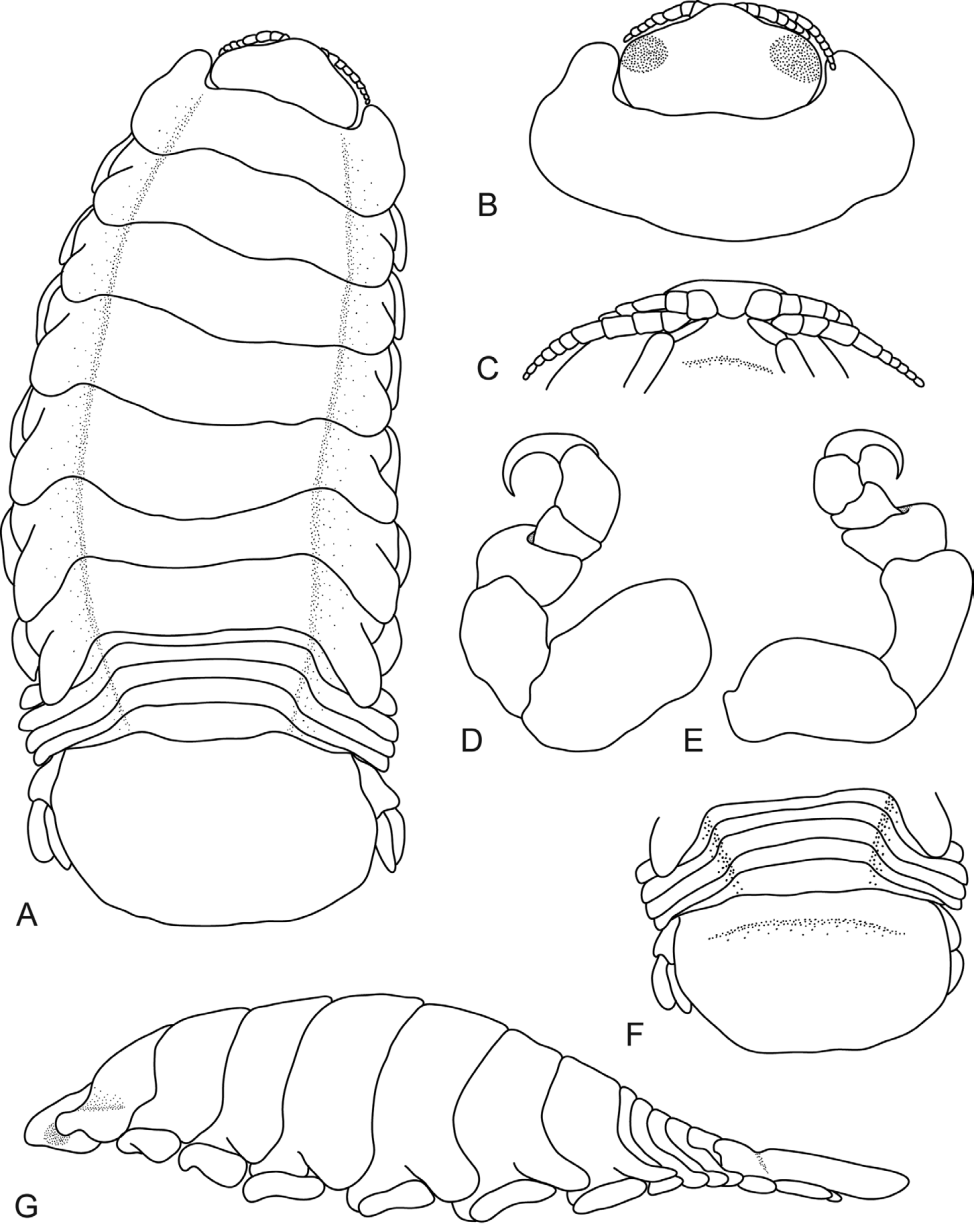
*Elthusa parva* (Richardson, 1910), comb. n., female holotype (19 mm) (USNM 40938). **A** dorsal view **B** dorsal view of pereonite 1 and cephalon **C** ventral view of cephalon **D** pereopod 1 **E** pereopod 7 **F** dorsal view of pleotelson **G** lateral view.

### *Ceratothoa* species list

A number of recent papers revising *Ceratothoa* (Martin et al. 2013, 2015a, Hadfield et al. 2014a, 2014b), has resulted in a number of changes to the accepted species in the genus. Several species are no longer valid and some have been placed into synonymy with other species. Currently accepted species of *Ceratothoa* are listed in Table 1; previous *Ceratothoa* combinations, showing the current status of the name as well as the earliest reference, are listed in Table 2.

**Table 1.** Currently accepted species of *Ceratothoa*, their respective authorities and the most recent reference for each.

No.	Accepted name	Authority	Reference
**1**	*Ceratothoa africanae*	Hadfield, Bruce & Smit, 2014	Hadfield et al. 2014b
**2**	*Ceratothoa angulata*	(Richardson, 1910b)	Present study
**3**	*Ceratothoa arimae*	(Nunomura, 2001)	Martin et al. 2015b
**4**	*Ceratothoa banksii*	(Leach, 1818)	Martin et al. 2015a
**5**	*Ceratothoa barracuda*	Martin, Bruce & Nowak, 2015	Martin et al. 2015a
**6**	*Ceratothoa capri*	(Trilles, 1964c)	Present study
**7**	*Ceratothoa carinata*	(Bianconi, 1869)	Present study
**8**	*Ceratothoa collaris*	Schioedte & Meinert, 1883	Present study
**9**	*Ceratothoa famosa*	Hadfield, Bruce & Smit, 2014	Hadfield et al. 2014b
**10**	*Ceratothoa gilberti*	(Richardson, 1904)	Present study
**11**	*Ceratothoa globulus*	Martin, Bruce & Nowak, 2015	Martin et al. 2015a
**12**	*Ceratothoa gobii*	Schioedte & Meinert, 1883	Present study
**13**	*Ceratothoa guttata*	(Richardson, 1910b)	Present study
**14**	*Ceratothoa imbricata*	(Fabricius, 1775)	Hadfield et al. 2014
**15**	*Ceratothoa italica*	Schioedte & Meinert, 1883	Present study
**16**	*Ceratothoa marisrubri*	Trilles, Colorni & Golani, 1999	Present study
**17**	*Ceratothoa oestroides*	(Risso, 1826)	Present study
**18**	*Ceratothoa oxyrrhynchaena*	Koelbel, 1878	Present study
**19**	*Ceratothoa parallela*	(Otto, 1828)	Present study
**20**	*Ceratothoa retusa*	(Schioedte & Meinert, 1883)	Hadfield et al. 2014a
**21**	*Ceratothoa steindachneri*	Koelbel, 1878	Present study
**22**	*Ceratothoa trigonocephala*	(Leach, 1818)	Hadfield et al. 2014b
**23**	*Ceratothoa usacarangis*	(Avdeev, 1979a)	Present study
**24**	*Ceratothoa verrucosa*	(Schioedte & Meinert, 1883)	Present study

**Table 2.** Species previously placed in combination with *Ceratothoa*, together with the current status and most recent reference. Full synonymies and nomenclatural details may be found in Trilles (1994).

No.	Former combination	Status	Reference
**1**	*Ceratothoa argus* (Haswell, 1881)	*nomen dubium* (no type, immature specimen)	Present study
**2**	*Ceratothoa atherinae* (Gourret, 1891)	= *Mothocya epimerica* (junior synonym)	Monod 1923c
**3**	*Ceratothoa brachyura* (White, 1847)	*nomen nudum* (no type, no description)	Present study
**4**	*Ceratothoa contracta* (Miers, 1880)	*species inquirenda* (type not located)	Martin et al. 2015a
**5**	*Ceratothoa crassa* Dana, 1853	*Glossobius crassa*	Stebbing 1893
**6**	*Ceratothoa curvicauda* Nunomura, 2006	= *Ceratothoa carinata* (junior synonym)	Martin et al. 2013
**7**	*Ceratothoa deplanata* Bovallius, 1885	= *Ceratothoa parallela* (junior synonym)	Horton 2000
**8**	*Ceratothoa directa* (Otto, 1821)	= *Ceratothoa parallela* (junior synonym)	Horton 2000
**9**	*Ceratothoa exocoeti* Cunningham, 1871	= *Glossobius impressus* (junior synonym)	Bruce and Bowman 1989
**10**	*Ceratothoa gaudichaudii* (Milne Edwards, 1840)	*species inquirenda* (no female type)	Martin et al. 2015
**11**	*Ceratothoa hemirhamphi* (Pillai, 1954)	= *Ceratothoa retusa* (junior synonym)	Bruce and Bowman 1989
**12**	*Ceratothoa huttoni* Filhol, 1885	= *Ceratothoa imbricata* (junior synonym)	Martin et al. 2015a
**13**	*Ceratothoa impressa* Say, 1818	= *Glossobius impressus* (junior synonym)	Bruce and Bowman 1989
**14**	*Ceratothoa laticauda* Milne Edwards, 1840	= *Glossobius auritus* (junior synonym)	Bruce and Bowman 1989
**15**	*Ceratothoa linearis* Dana, 1853	*Glossobius linearis*	Stebbing 1893
**16**	*Ceratothoa lineata* Miers, 1876	*Mothocya lineata*	Martin et al. 2015a
**17**	*Ceratothoa novae–zelandiae* Filhol, 1885	= *Ceratothoa trigonocephala* (junior synonym)	Trilles 1972b
**18**	*Ceratothoa parva* (Richardson, 1910b)	*incertae sedis* (not *Ceratothoa*, single specimen)	Present study
**19**	*Ceratothoa potassoniensis* (Penso, 1939)	*nomen dubium* (no type or description)	Present study
**20**	*Ceratothoa poutassouiensis* (Brian, 1939)	*nomen nudum* (no type or description)	Horton 2000
**21**	*Ceratothoa rapax* Heller, 1865	= *Ceratothoa gaudichaudii* (junior synonym)	Schioedte and Meinert 1883
**22**	*Ceratothoa salparum* Gourret, 1891	= *Emetha audouini* (junior synonym)	Trilles 1972a
**23**	*Ceratothoa sargorum* Gourret, 1891	= *Ceratothoa oestroïdes* (junior synonym)	Radujković et al. 1984
**24**	*Ceratothoa transversa* (Richardson, 1901)	*species inquirenda* (immature specimen)	Present study
**25**	*Ceratothoa triglae* Gourret, 1891	*species inquirenda* (no type, male specimen)	Present study
**26**	*Ceratothoa trillesi* (Avdeev, 1979a)	= *Ceratothoa imbricata* (junior synonym)	Martin et al. 2015a
**27**	*Ceratothoa venusta* Avdeev, 1978	= *Ceratothoa guttata* (junior synonym)	Bruce and Bowman 1989

## Conclusion

A total of 50 *Ceratothoa* species names were found in the literature. Of these, 30 are regarded as valid (Bruce and Schotte 2016). Following recent work (Martin et al. 2013, 2015a, Hadfield et al. 2014b) and this present study, we accept 24 species of *Ceratothoa* as valid. This review of the poorly studied *Ceratothoa* resolves some of the uncertainties surrounding certain species and provides an updated list of valid *Ceratothoa* species.

## Supplementary Material

XML Treatment for Ceratothoa

XML Treatment for Ceratothoa angulata

XML Treatment for Ceratothoa capri

XML Treatment for Ceratothoa carinata

XML Treatment for Ceratothoa collaris

XML Treatment for Ceratothoa gilberti

XML Treatment for Ceratothoa gobii

XML Treatment for Ceratothoa guttata

XML Treatment for Ceratothoa italica

XML Treatment for Ceratothoa oestroides

XML Treatment for Ceratothoa verrucosa

XML Treatment for Ceratothoa argus

XML Treatment for Ceratothoa poutassouiensis

XML Treatment for Ceratothoa transversa

XML Treatment for Ceratothoa triglae

XML Treatment for Elthusa parva
